# CENP-A overexpression promotes distinct fates in human cells, depending on p53 status

**DOI:** 10.1038/s42003-021-01941-5

**Published:** 2021-03-26

**Authors:** Daniel Jeffery, Alberto Gatto, Katrina Podsypanina, Charlène Renaud-Pageot, Rebeca Ponce Landete, Lorraine Bonneville, Marie Dumont, Daniele Fachinetti, Geneviève Almouzni

**Affiliations:** 1 Institut Curie, PSL Research University, CNRS, Sorbonne Université, Nuclear Dynamics Unit, Equipe Labellisée Ligue contre le Cancer, Paris, France; 2 Institut Curie, PSL Research University, Centre de Recherche, Sorbonne Université, Cell Biology and Cancer Unit, Paris, France

**Keywords:** Cancer therapeutic resistance, Epithelial-mesenchymal transition, Cell-cycle exit, Centromeres

## Abstract

Tumour evolution is driven by both genetic and epigenetic changes. CENP-A, the centromeric histone H3 variant, is an epigenetic mark that directly perturbs genetic stability and chromatin when overexpressed. Although CENP-A overexpression is a common feature of many cancers, how this impacts cell fate and response to therapy remains unclear. Here, we established a tunable system of inducible and reversible CENP-A overexpression combined with a switch in p53 status in human cell lines. Through clonogenic survival assays, single-cell RNA-sequencing and cell trajectory analysis, we uncover the tumour suppressor p53 as a key determinant of how CENP-A impacts cell state, cell identity and therapeutic response. If p53 is functional, CENP-A overexpression promotes senescence and radiosensitivity. Surprisingly, when we inactivate p53, CENP-A overexpression instead promotes epithelial-mesenchymal transition, an essential process in mammalian development but also a precursor for tumour cell invasion and metastasis. Thus, we uncover an unanticipated function of CENP-A overexpression to promote cell fate reprogramming, with important implications for development and tumour evolution.

## Abstract (web summary)

Jeffery et al. develop a system of inducible and reversible CENP-A overexpression, combined with a switch in p53 status in human cell lines. They find the tumor suppressor p53 as a key determinant of how CENP-A impacts cell state, cell identity and therapeutic response. This study suggests that CENP-A overexpression promotes the reprogramming of cell fates in the context of tumor evolution.

## Introduction

Tumor evolution is driven by both genetic and nongenetic changes from tumourigenesis to therapeutic resistance^1,2^. The fate of a given cell depends on how these changes impact both cell state (e.g., proliferation or death) and cell identity (e.g., stemness or differentiation). On the genetic side, tumor evolution is strongly linked to chromosomal instability (CIN)^3–5^. This subcategory of genome instability is characterized by an increased rate of mutagenesis through losses or gains of chromosomes, partial chromosomes, or chromosomal rearrangements. While extreme CIN is generally deleterious to cells, low or intermediate CIN increases the heterogeneity of the cell population^6^. These changes can promote the selection of advantageous clones through Darwinian evolution^7^ a hypothesis put forward more than a century ago in cancer biology^8^. On the nongenetic side, environmental and metabolic insults can induce changes to epigenetic landscapes that perturb genome regulation^9^. This can further contribute to tumor heterogeneity^10^ enabling cell plasticity^11^, acquisition and maintenance of stemness properties^12^, and the ability to counteract cell shutdown mechanisms^9^. All of these aspects can promote tumor development and reduce therapeutic response. But, in contrast to genetic changes, epigenetic changes are reversible^13^. This reversibility makes epigenetic marks and their regulatory factors interesting targets for cancer treatment, especially in combination with other anticancer therapies^14^.

Furthermore, genetic and epigenetic parameters are tightly interconnected. As an illustration, the centromeric histone H3 variant, centromere protein A (CENP-A), is an epigenetic mark with direct effects on CIN^15^. In mammals, CENP-A is considered as a prime example of a bona fide transgenerational epigenetic mark^16^. Indeed, its deposition determines the location of the centromere^17,18^ and it is transmitted across cell divisions and even organismal generations^19,20^. CENP-A is deposited^21,22^ and maintained^23^ at centric chromatin by its dedicated histone chaperone HJURP. CENP-A acts as the foundation for kinetochore assembly during cell division, where it is essential for efficient chromosome segregation (reviewed in^24–27^). Therefore, the maintenance of genome integrity depends on the proper regulation of CENP-A^28^. This is demonstrated by CENP-A depletion^29^ and overexpression^30^ experiments, which both promote mitotic defects and CIN in human cells. Importantly, high CENP-A levels correlate strongly with increased tumor aggressiveness in patients^31–39^, including an intriguing connection with increased invasiveness/metastasis^31,33,34,36,37,39^. Examining the regulation of *CENPA* gene expression, we demonstrated that the tumor suppressor p53 (reviewed in^40,41^) negatively regulates CENP-A transcription through its downstream effector p21^42^. Thus, CENP-A levels are held in check by active p53. But CENP-A overexpression occurs in many cancers^36,37^, and although *TP53* mutation correlates with higher CENP-A levels in patient tumors^42^, many tumors overexpress CENP-A despite having wild-type (WT) *TP53* (cBioPortal^43^), indicating that p53 regulation is not the sole mechanism that controls CENP-A expression. Notably, correlation of high CENP-A levels with therapeutic response to DNA damaging agents is a matter of debate, with studies arguing for reduced^31^ or improved response^37,39^. However, p53 status was not assessed in these studies. This is even more important, given that the p53 pathway responds differentially to distinct cell cycle defects^44^. Thus, understanding how CENP-A overexpression impacts cell fate in different p53 contexts is an important question that could shed light on how CENP-A influences tumor evolution, including tumor invasiveness and metastasis as well as cell response to cancer treatment.

To address these issues, we set up a system to turn on and off the overexpression of CENP-A in various p53 contexts and switch p53 status in selected cell lines. Using this tunable system, we demonstrate that CENP-A overexpression alters cell fate in a manner dependent on p53 status. When p53 is functional, CENP-A overexpression alters cell state, promoting cell cycle arrest, senescence, and radiosensitivity. But when we inactivate p53 the cells evade arrest. Instead, CENP-A overexpression stimulates a change in cell identity, promoting epithelial–mesenchymal transition (EMT). Thus, our findings reveal an unanticipated function of CENP-A overexpression to promote the reprogramming of cell fate in distinct ways that depend on p53 status, with important implications for the role of CENP-A in tumor evolution.

## Results

### Inducible and reversible CENP-A overexpression in cells with varied p53 status

**Fig. 1:**
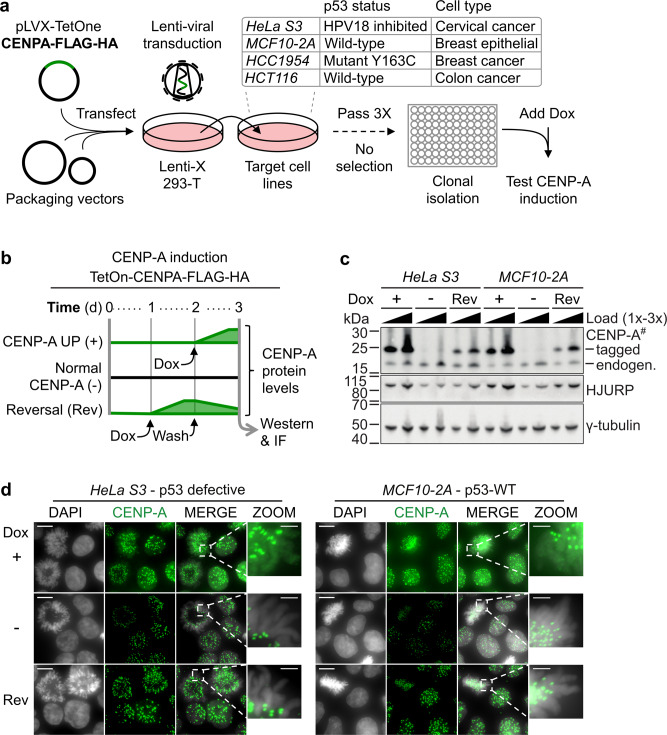
Inducible and reversible CENP-A overexpression in cells with varied p53 status. **a** Scheme for the generation of doxycycline (Dox) inducible CENP-A overexpression cell lines. *TetOn-CENPA-FLAG-HA* construct was randomly integrated into several indicated target cell lines by lentiviral transfection without selection. After clonal isolation, we tested cells for clear homogenous CENP-A and HA increase by IF in approximately all cells 24 h after the addition of 80 ng/ml of Dox. Cells were also tested to ensure no detectable background HA signal by western or IF, when no Dox was added. **b** Scheme representing relative CENP-A protein levels over time for *TetOn-CENPA-FLAG-HA* cell lines. Dox = 10 ng/ml. **c** Western blot of total cell extracts (TCEs) pertaining to scheme in (**b**). Primary antibodies indicated on the right. Exogenous CENP-A (tagged) can be distinguished from endogenous CENP-A (endogen.) by its increased molecular weight. # = high sensitivity ECL. y-tubulin used as loading control. See also Supplementary Fig. 1B for western blot analysis corresponding to all cell lines. **d** Immunofluorescence of paraformaldehyde-fixed cells using anti-CENP-A antibody (green) and DAPI staining (gray). Conditions in parallel with (**c**). Showing max projection images from a Z-series with zoom on a mitotic cell (DAPI/CENP-A merge) to highlight chromosome arms. Scale bars = 10 µm, Zoom = 2.5 µm.

In order to explore how CENP-A expression levels and p53 status impact cell fate and cell response to anticancer treatment, we first established a system where we could specifically alter CENP-A expression independent of its regulation by p53. For this, we added a doxycycline-inducible CENP-A overexpression construct (*TetOn-CENPA-FLAG-HA*) by lentiviral transduction into several cell lines with varied p53 status (Fig. 1a, see also Table 1). Importantly, we did not use antibiotic selection to obtain CENP-A overexpression. This enables us to investigate the unbiased effects of CENP-A overexpression over time, without provoking cell adaptations or secondary mutations that could arise from selective pressure. The TetOn system allows induction of CENP-A overexpression within 24 h of adding doxycycline (Fig. 1b–d). This induction is within the range of CENP-A levels found in patient tumors, which can go over 1000-fold relative to healthy tissue (Supplementary Fig. 1A, B; cBioPortal^43^). Even in healthy tissues, there is a high degree of variability in CENP-A expression, where differences up to 50-fold can be observed (cf. CENP-A tissue expression in www.proteinatlas.org^45^). This acute-induced overexpression of CENP-A increases HJURP protein levels (Fig. 1c), increases levels of CENP-A at centromeres, and also causes mislocalization of CENP-A to the chromosome arms (Fig. 1d), regardless of p53 status. These effects are similar to those observed with constitutive CENP-A overexpression in HeLa cells^46^. Here, however, we could reverse both CENP-A overexpression and mislocalization simply by washing out doxycycline (Fig. 1c, d), consistent with the observation of rapid removal of ectopic CENP-A from chromosome arms with DNA replication^47^. Thus, while artificial, our controlled system is a powerful tool to test therapeutic sensitivity in several cell lines with varied p53 status under conditions of tunable CENP-A overexpression within the range of expression found in tumors.

**Table 1:** Description of cell lines used and their p53 status.

Cell line	p53 status^a^	Cell type	Construct	Comments
MCF10-2A	Functional wildtype	Immortal non-tumoral (fibrocystic disease) breast epithelial	*TetOn-CENP-A-FLAG-HA* (lentiviral)	Female origin
HCT116	Functional wildtype	Colon cancer	*TetOn-CENP-A-FLAG-HA* (lentiviral)	Male origin
HeLa S3	Nonfunctional wildtype: HPV18 inactivated	Cervical cancer	*TetOn-CENP-A-FLAG-HA* (lentiviral)	Female origin
HCC1954	LOF p53 mutation at Y163C	Invasive ductal carcinoma, breast	*TetOn-CENP-A-FLAG-HA* (lentiviral)	Female origin; HER^+^ ER^−^ PR^−^ luminal
DLD1	One functionally silent allele of p53 and LOF mutation at S241F	Colon cancer	*TetOn-CENPA-YFP-AID* (FlpIn)	Male origin; kind gift from D. Fachinetti

^a^p53 status for missense mutations from the TP53 database^107^.

### Induced CENP-A overexpression causes reversible radiosensitivity in p53-WT cells

**Fig. 2:**
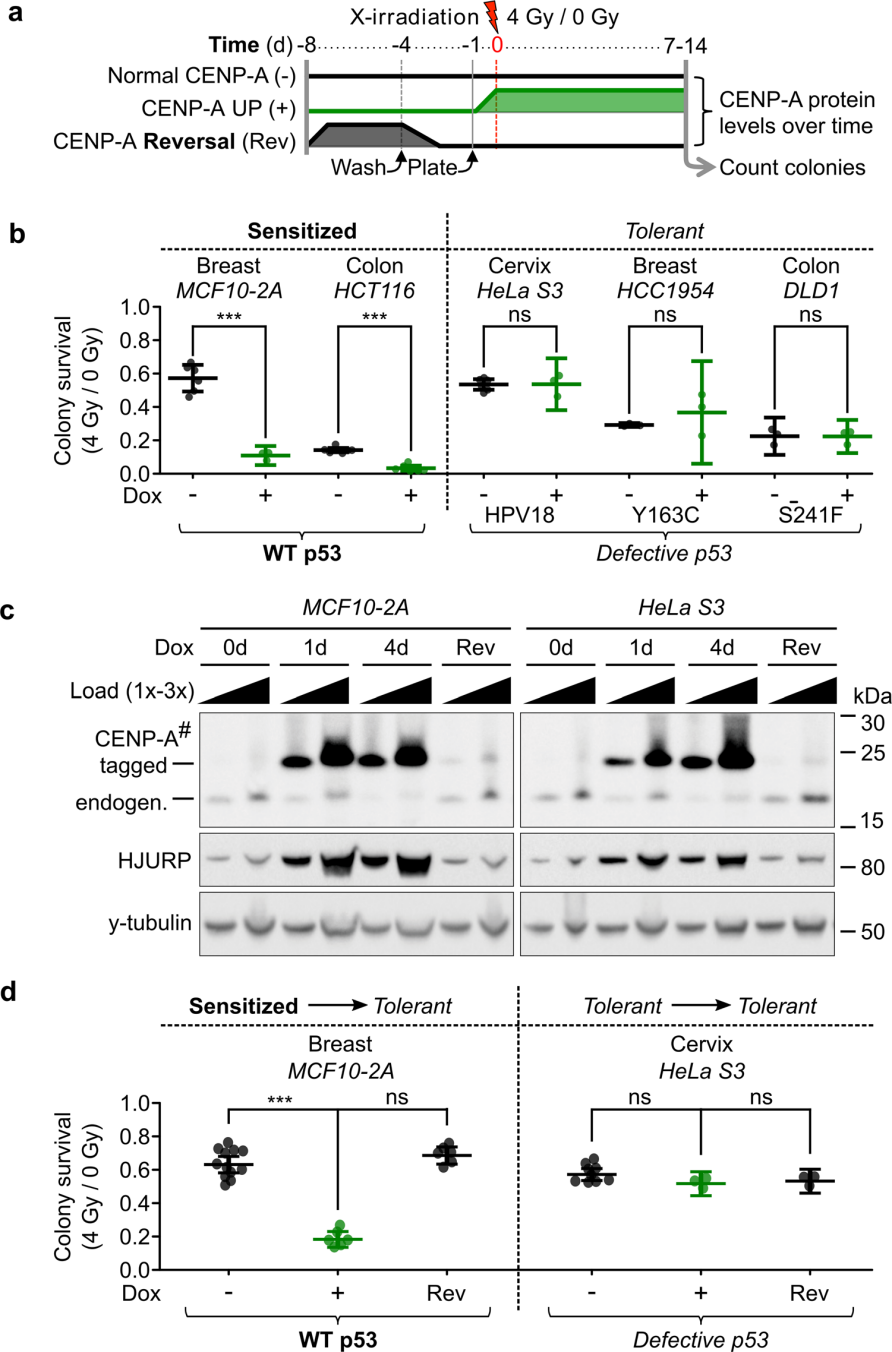
Induced CENP-A overexpression causes reversible radiosensitivity in p53 wild-type cells. **a** Scheme of colony formation assays (CFAs) with relative CENP-A protein levels over time for *TetOn-CENPA* cell lines. **b** CFA results corresponding to the scheme in (**a**) for five different cell lines of varied tissue origins and p53 status, as indicated. Each dot represents a single biological replicate. Plots show mean and 95% confidence interval. Statistical significance tested by two-tailed Welch’s *t* test with Bonferroni cutoff at a *p* value of 0.01 (*α* = 0.05). *** = *p* value < 0.0001. See also Supplementary Fig. 1C for survival ratios relative to untreated condition. **c** Western blot corresponding to scheme in (**a**): TCEs after 1 day (1d) or 4d with 10 ng/ml Dox, no Dox control (0d), or 4d Dox followed by 4d without Dox (Rev). Primary antibodies indicated on the left. 1x load = ~30,000 cells. # = high sensitivity ECL exposure. y-tubulin used as loading control. **d** CFA results corresponding to scheme in (**a**) for the indicated cell lines. Plots as in (**b**). Statistical significance tested by two-tailed Welch’s *t* test, compared to non-induced control, with Bonferroni cutoff at a *p* value of 0.0125 (*α* = 0.05). *** = *p* value < 0.0001.

To test therapeutic sensitivity in our cell lines, we chose X-irradiation as a representative DNA damaging agent that is commonly used in cancer therapy. Thus, we tested cell survival after X-irradiation with or without induction of CENP-A overexpression by colony formation assays (CFAs), a surrogate for self-renewal capacity (Fig. 2a). For cell lines with WT p53 status, we used both non-tumoral (MCF10-2A) and tumoral (HCT116) lines. CENP-A overexpression in both cell lines led to a strong increase in sensitivity to X-irradiation, independent of tumoral status (Fig. 2b, left). Meanwhile, CENP-A overexpression in all cell lines with defective p53 (HeLa S3, HCC1954, and DLD1) did not significantly affect radiosensitivity—a distinctly radio-tolerant phenotype (Fig. 2b, right, see also Supplementary Fig. 1C). To assess if these distinct CFA phenotypes could be explained by the different CENP-A levels associated with each cell line, we first compared by western blotting the levels of endogenous CENP-A and CENP-A overexpression in all cell lines used (Supplementary Fig. 1B). Since our data do not show a correlation between CENP-A levels and radiosensitivity upon CENP-A overexpression, we can discard this explanation. Second, in order to determine if the differences in sensitivity that we observed upon CENP-A overexpression were due to secondary mutations from mitotic defects, we tested the reversibility of this sensitivity by tuning down CENP-A expression to near-endogenous levels in p53-WT cells (MCF10-2A) and p53-defective cells (HeLa S3) (Fig. 2c). Remarkably, restoration of CENP-A expression to endogenous levels reversed the sensitivity phenotype in MCF10-2A, while HeLa cells, used here as a control line, remained tolerant (Fig. 2d). Therefore, the radiosensitivity observed in MCF10-2A cells requires the continued overexpression of CENP-A and it cannot be explained by genetic effects (i.e., secondary mutations) that occur after induced CENP-A overexpression. Thus, CENP-A overexpression in both tumoral and non-tumoral cell lines leads to radiosensitivity in a manner that correlates with p53 status.

### Sensitivity to X-irradiation upon CENP-A overexpression is dependent on functional p53

**Fig. 3:**
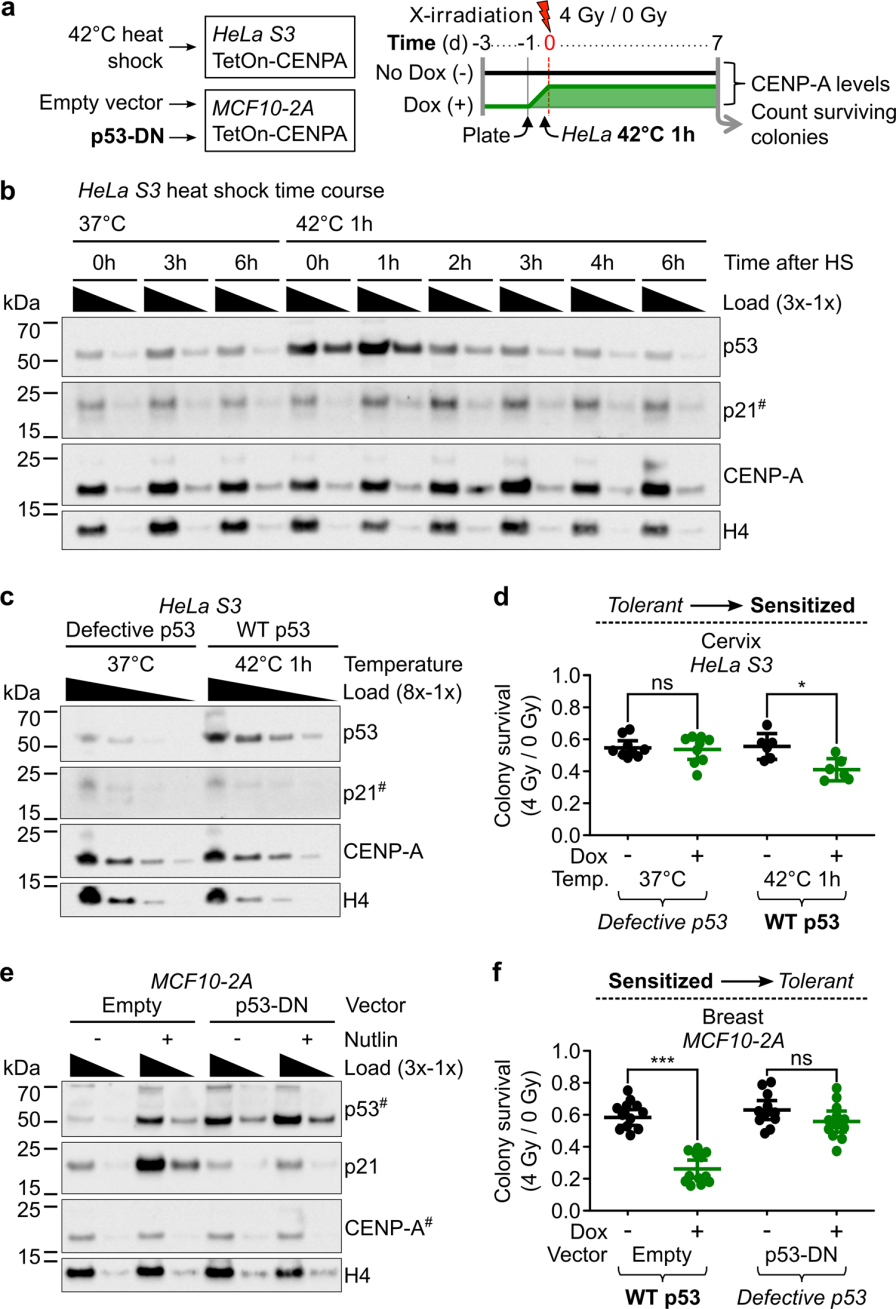
Sensitivity to X-irradiation upon CENP-A overexpression is dependent on functional p53. **a** Scheme of colony formation assays (CFAs) and change of p53 status with relative CENP-A protein levels over time for *TetOn-CENPA-FLAG-HA* cell lines. WT p53 is transiently activated in HeLa cells by a short heat shock, while p53 is stably inactivated in MCF10-2A cells by expression of a p53 dominant-negative peptide (p53-DN). **b** Temporary change of p53 status in HeLa cells by 1 h heat shock (HS). Western blot of HeLa cell TCEs at indicated time points following 1 h at 42 or 37 °C control. Temporary increase of p53 and subsequently p21. 1x load = ~33,000 cells. Primary antibodies are indicated on the right. ^#^ = high sensitivity ECL exposure. H4 used as loading control. **c** Increase of p53 levels in HeLa cells by heat shock. Western blot of TCEs after 1 h at 42 or 37 °C control. Loaded at 8x, 4x, 2x, 1x concentrations, where 1x load = ~12,500 cells. Primary antibodies are indicated on the right. ^#^ = high sensitivity ECL exposure. H4 used as loading control. Representative western blot from three independent experiments is shown. **d** HeLa S3 CFAs pertaining to scheme in (**a**). Each dot represents a single biological replicate. Plots show mean and 95% confidence interval. Statistical significance tested by two-tailed Welch’s *t* test with Bonferroni cutoff at a *p* value of 0.0125 (*α* = 0.05). * = *p* value < 0.01. **e** Change of p53 status in *MCF10-2A TetOn-CENPA-FLAG-HA* cells. Western blot of TCEs after 24 h of 10 µM Nutlin-3 treatment or DMSO control, as indicated. Dominant negative p53 peptide stabilizes p53 and suppresses its activation of p21. 1x load corresponds to total protein extract from ~30,000 cells. Primary antibodies are indicated on the right. ^#^ = high sensitivity ECL exposure. H4 used as loading control. Representative western blot from three independent experiments is shown. **f** MCF10-2A CFAs pertaining to scheme in (**a**). Each dot represents a single biological replicate. Plots show mean and 95% confidence interval. Statistical significance tested by two-tailed Welch’s *t* test with Bonferroni cutoff at a *p* value of 0.0125 (*α* = 0.05). *** = *p* value < 0.0001. See also Supplementary Fig. 1C, D for CFAs following range of Dox and X-irradiation doses and Supplementary Fig. 1E for representative CFA images.

To assess if p53 plays a causal role in the radiosensitivity associated with CENP-A overexpression, we took advantage of the possibility to switch the p53 status in two of our inducible CENP-A overexpression cell lines: the tolerant HeLa cells and the sensitized MCF10-2A cells. We could thus carry out CFAs after CENP-A overexpression and X-irradiation in isogenic cell lines, but with altered p53 status (Fig. 3a). For the change from p53-defective to p53-WT in the CENP-A inducible HeLa cells, we transiently activated WT p53 by incubation at 42 °C for 1 h immediately prior to X-irradiation. This treatment enables the temporary release of p53 repression from the HPV18 E6 protein, permitting a functional p53 response^48^. We confirmed that after hyperthermia treatment, p53 levels increased, followed shortly after by increased p21 (Fig. 3b, c). This short hyperthermia treatment in HeLa cells was sufficient to mildly, but significantly, increase sensitivity to X-irradiation after CENP-A overexpression (Fig. 3d). The fact that we find an increase in sensitivity only when CENP-A is overexpressed is consistent with the hypothesis of an effect dependent on the transient reactivation of p53, however, we cannot exclude potential effects of hyperthermia independent of p53. In this regard, it was important to specifically alter only the p53 status in a controlled manner. For this, we stably transduced the p53-WT MCF10-2A *TetON-CENPA-FLAG-HA* cells with either an empty vector control or a constitutively expressed dominant negative (DN) *TP53* construct, in order to mimic p53 loss and p53 loss of function (LOF) mutations (p53DD construct^49^). The p53-DN peptide stabilizes the WT p53 protein, causing a clear increase in p53 protein level, but suppresses its activation of downstream targets (e.g., p21). Indeed, the indirect activator of p53 (Nutlin-3) caused clear increases in p53 and p21 in p53-WT cells, but not those expressing the p53-DN peptide (Fig. 3e). By stably switching p53 status in this manner, we observed a major change in the radiosensitivity of the cells. The p53-DN peptide in MCF10-2A cells strongly counteracted the radiosensitivity phenotype associated with CENP-A overexpression (Fig. 3f). Furthermore, we confirmed the radiosensitivity phenotype using a range of doxycycline concentrations and a range of X-irradiation doses in CFAs for the p53-WT and p53-DN MCF10-2A cells (Supplementary Fig. 1C, D). Taken together, the results demonstrate that induced CENP-A overexpression increases radiosensitivity in a p53-dependent manner.

### Induced CENP-A overexpression promotes radiosensitivity by impairing cell cycle progression

**Fig. 4:**
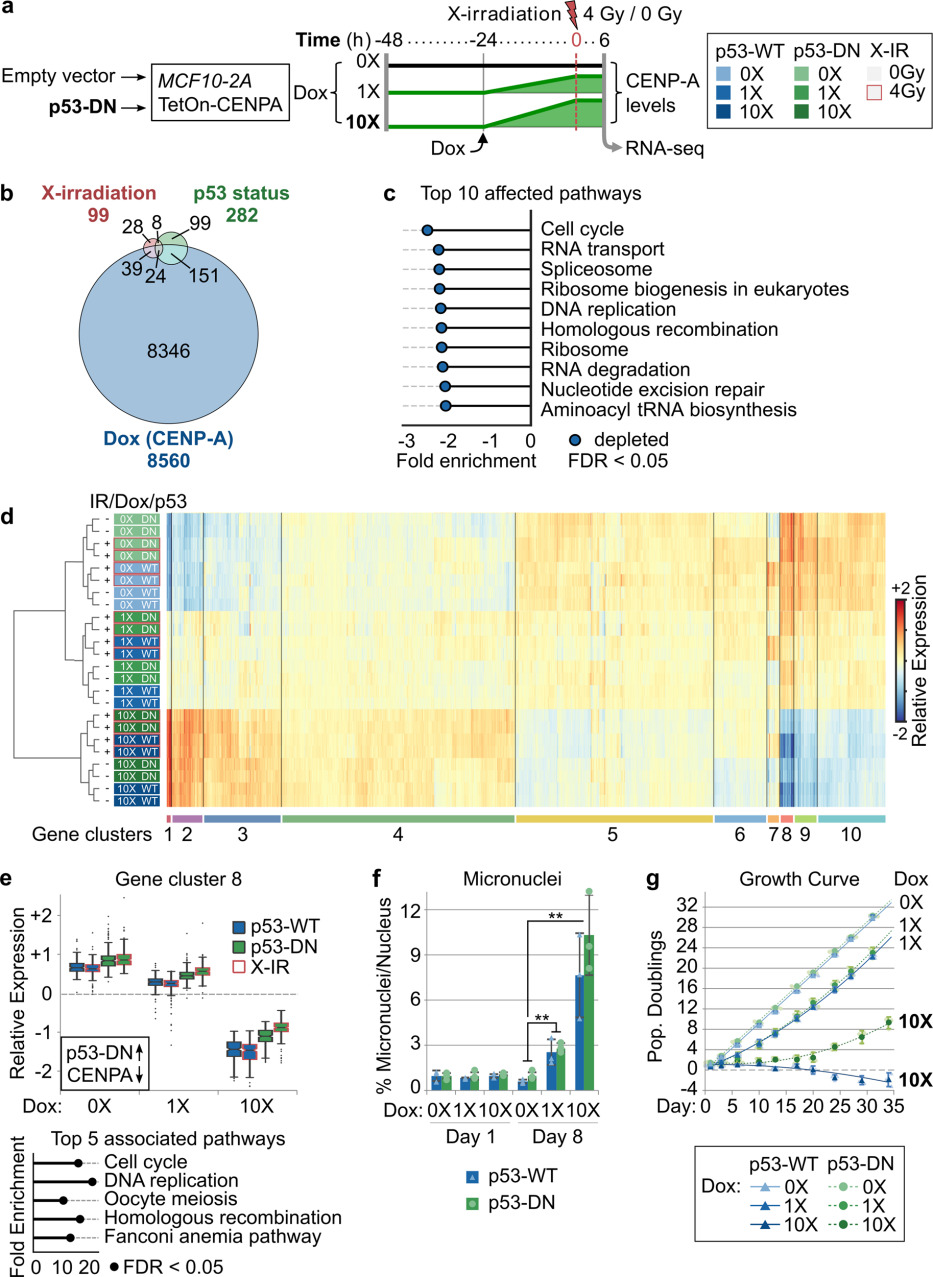
Induced CENP-A overexpression promotes radiosensitivity by impairing cell cycle progression. **a** Scheme delineating conditions tested by RNA-sequencing showing relative CENP-A protein levels over time and corresponding legend (pertains to (**d**)). All conditions tested in duplicate. See Supplementary Fig. 1F for CENP-A protein levels corresponding to 0X, 1X (10 ng/ml), and 10X (100 ng/ml) Dox. The transcriptional impact of Dox treatment alone was tested in the non-inducible MCF10-2A parental control (see Supplementary Fig. 2A, B). **b** Proportional Venn diagram summarizing the number of differentially expressed genes (DEGs) upon X-irradiation (red, 0 vs 4 Gy); change of p53 status (green, p53-WT vs p53-DN); and CENP-A overexpression (blue, 0X Dox vs 1X Dox vs 10X Dox). Number of DEGs in each category and overlapping categories is indicated. **c** Gene set enrichment analysis showing the top 10 KEGG pathways affected by CENP-A overexpression (WebGestaltR v0.4.2) and normalized enrichment score. All ten pathways are downregulated with increasing levels of Dox (i.e., significant depletion, at FDR < 0.05). See Supplementary Data 2 (GSEA) for all significantly depleted/enriched processes. **d** Heat map showing hierarchical clustering of samples (rows, colored according to legend in (**a**)) and DEGs (columns), with the ten main gene clusters annotated below. Relative expression: expression relative to the average for a given gene across all conditions (mean-centered counts, log_2_ transformed, and TMM normalized). See also Supplementary Data 2 for normalized counts, differential expression analysis, and cluster assignment for all genes and conditions. **e** Top: box plot showing the distribution of expression levels for DEGs in cluster 8 according to experimental condition. Center lines show the medians; box limits indicate the 25th and 75th percentiles; whiskers extend 1.5 times the interquartile range from the 25th and 75th percentiles; outliers represented by dots. See Supplementary Fig. 2C for all gene clusters. Black box: for genes in this cluster, p53-DN increases expression (up arrow) and CENP-A overexpression decreases expression (down arrow). Bottom: top 5 enriched KEGG pathways (WebGestaltR v0.4.2) for genes within cluster 8 based on overrepresentation analysis (ORA). All five terms are significantly overrepresented (FDR < 0.05). See Supplementary Data 2 (ORA) for top 10 pathways for all clusters. **f** Accumulation of micronuclei pertaining to days 1 and 8 of MCF10-2A *TetOn-CENPA-FLAG-HA* cells with either empty vector (p53-WT) or dominant-negative p53 (p53-DN) grown continuously with 0X Dox, 1X Dox (10 ng/ml), or 10X Dox (100 ng/ml). Plots show mean and 95% confidence interval for three biological replicates (triangles/circles) from a single experiment. Statistical significance tested by two-tailed Welch’s *t* test with Bonferroni cutoff at a *p* value of 0.01 (*α* = 0.05). No significant differences between p53-WT and p53-DN samples with same Dox treatment. ** = *p* value < 0.001. *N* = > 1000 nuclei per condition for each replicate. **g** Growth curve of MCF10-2A *TetOn-CENPA-FLAG-HA* cells with either empty vector (p53-WT) or dominant-negative p53 (p53-DN) grown continuously with 0X Dox, 1X Dox (10 ng/ml), or 10X Dox (100 ng/ml). Growth curve shows number of population doublings relative to the initial seeding population at day −1. Circles/triangles show mean and 95% confidence interval for three biological replicates (smaller circles/triangles) at each time point where cells were counted. Lines show best-fit curves. Similar results were obtained for a second independent experiment (see Supplementary Fig. 3G for growth curve of the non-inducible parental MCF10-2A cells after Dox exposure).

Given that the p53-dependent radiosensitivity was due to nongenetic (reversible) effects of CENP-A overexpression, we decided to assess changes at the transcriptional level. We first assessed global transcription by RNA-seq, analyzing the effects of acute CENP-A overexpression (at two different concentrations of Dox), switch of p53 status, and X-irradiation treatment in the MCF10-2A cells (Fig. 4a). X-irradiation and inactivation of p53 affected the transcription of several genes (Fig. 4b). Notably, CENP-A overexpression induced more extensive changes, with more than 8000 genes affected. According to gene set enrichment analysis (GSEA), CENP-A overexpression mainly led to the repression of genes involved in cell cycle progression, DNA repair, and RNA metabolism (Fig. 4c, details in Supplementary Data 2). To more specifically identify the key cellular pathways involved in radiosensitivity and its reversal by inactivation of p53, we applied hierarchical clustering to identify co-regulated subsets of differentially expressed genes (DEGs). In particular, we wanted to identify genes for which p53-DN could counteract the effects of CENP-A overexpression. Out of ten clusters (Fig. 4d, see also Supplementary Fig. 2C), there was only one that corresponded to these criteria (cluster 8). This subset of genes is strongly downregulated by CENP-A overexpression and upregulated by p53-DN (Fig. 4e). Importantly, this upregulation is further increased after X-irradiation and these effects are significant even at the lower dose of CENP-A overexpression (see Supplementary Data 2 for significance testing). Furthermore, we verified whether the effect of CENP-A overexpression on these genes also extends to cancer cell lines with different genetic backgrounds. We performed reverse transcription quantitative PCR of selected cluster 8 genes after CENP-A overexpression in the p53-WT HCT116 and p53-defective DLD1 colon cancer cell lines for comparison. CENP-A overexpression decreased the relative expression of this group of genes in the p53-WT HCT116 cell line, and had a somewhat reduced effect in the p53-defective DLD1 cells (Supplementary Fig. 2D). The fact that these data are consistent with the effects observed in MCF10-2A p53-WT and p53-DN cells further emphasizes the importance of this core set of genes. Overrepresentation analysis (ORA) shows that these genes are mainly involved in cell cycle control, followed by DNA repair (Fig. 4e, bottom). We then used cellular assays to investigate the effects of CENP-A overexpression and p53 inactivation on these two pathways. Concerning DNA repair, we did not detect significant changes in either acute DNA damage or rate of DNA repair (Supplementary Fig. 3A–D). However, on a longer timescale, we observed an increase in mitotic stress associated with CENP-A overexpression that was similar in both p53-WT and p53-DN cells (micronuclei in Fig. 4f, and CIN in Supplementary Fig. 3E, including numerical and structural aneuploidy by multicolor fluorescence in situ hybridization (mFISH) karyotypes). This is in agreement with previous studies^30,50^. In the p53-WT cells, this mitotic stress was associated with p53 activation, as shown by western blot (Supplementary Fig. 3F). As for cell cycle control, CENP-A overexpression leads to prolonged growth inhibition, even in the absence of irradiation (Fig. 4g). This is consistent with the reduced colony size and colony density visible in the CFA stains (Supplementary Fig. 1D). Importantly, this growth inhibition is amplified with increased Dox and is partially counteracted by p53-DN (Fig. 4g, see also Supplementary Fig. 3G for control). These findings enabled us to discard a major role for DNA damage or repair in the radiosensitivity phenotype. Therefore, based on both the bulk RNA-seq data and cellular assays, we conclude that induced CENP-A overexpression leads to p53-dependent radiosensitivity mainly by impairing cell cycle progression. We then wished to understand how this was operating at the level of individual cells.

### CENP-A overexpression promotes acute cell cycle arrest and senescence in p53-WT cells

**Fig. 5:**
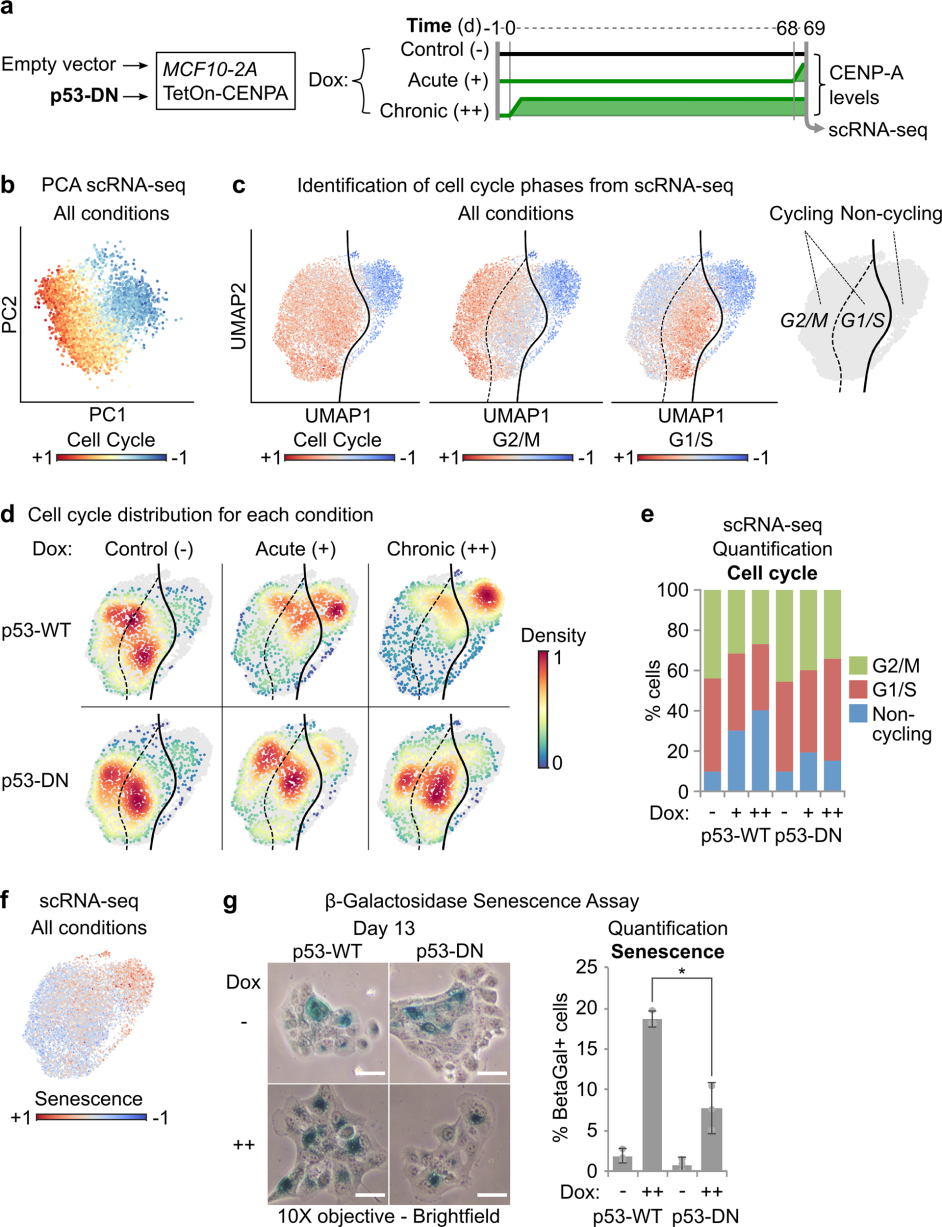
CENP-A overexpression promotes acute cell cycle arrest and senescence in p53-WT cells. **a** Scheme delineating conditions tested by single-cell RNA-sequencing showing relative CENP-A protein levels over time (pertaining to **b**–**f**, Fig. 6a–d, Supplementary Figs. 4B, C and 5A–D, and Supplementary Data 3). **b** Principal component analysis (PCA) of all scRNA-seq samples, illustrated in (**a**). All conditions merged. Each dot represents a single cell on the first two principal components (PC1 and PC2), colored by expression of cell cycle genes (cell cycle signature includes all genes from Cyclebase v3.0, *CENPA* excluded). **c** Uniform Manifold Approximation and Projection (UMAP) maps of scRNA-seq data, pertaining to scheme in (**a**). All conditions combined. Each dot corresponds to an individual cell projected in a 2D space where the topology reflects global similarities in expression. The color gradient is proportional to the expression of cell cycle, G2/M, and G1/S genes (Cyclebase v3.0). Far right plot: boundary between cycling (G2/M or G1/S) and non-cycling cells in UMAP space computed by support vector classification. **d** Cell density by condition (color gradient) in UMAP space for each scRNA-seq experimental condition, pertaining to scheme in (**a**). **e** Stacked bar plot showing the % of cells in G2/M, G1/S, and non-cycling scRNA-seq clusters for each experimental condition. **f** UMAP of scRNA-seq data as in (**c**) for all conditions merged, colored by expression gradient of senescence-associated genes (Fridman Senescence Up gene set from MSigDB v6.2). **g** Beta-galactosidase senescence assays. Left: representative brightfield images after 13 days of growth without Dox (−) or with chronic Dox (10 ng/ml, ++), taken with a 10X objective. Scale bars = 20 µm. Only cells with dark blue staining were considered senescent. Right: quantification of frequency of senescence, showing mean and 95% confidence interval from a total of three biological replicates (gray circles) across two independent experiments. *N* = > 100 cells per condition per replicate. * = two-tailed Welch’s *t* test comparing p53-WT to p53-DN after chronic CENP-A overexpression; *p* value = 0.014.

To determine the effects of CENP-A overexpression and p53 status on individual cell fate and evolution of the cell population over time, we performed single-cell RNA-sequencing (scRNA-seq), a powerful tool for characterizing cell state and identity^51^. Using our p53-WT and p53-DN MCF10-2A cells, we compared prolonged, continuous CENP-A overexpression (chronic induction for 69 days), to acute CENP-A overexpression (1 day) and non-overexpressing conditions, passaged in parallel (see scheme in Fig. 5a). Given that gene expression changes were detectable even at low doses of Dox (Fig. 4d and Supplementary Data 2), we chose this condition to avoid the cytotoxic effects of higher doses (Fig. 4f and Supplementary Fig. 1D). First, we confirmed the chronic overexpression of CENP-A by western blot (Supplementary Fig. 4A) and verified that the scRNA-seq data agreed with the bulk RNA-seq data from comparable samples (Supplementary Fig. 4B). Principle component analysis of the single-cell expression profiles revealed that most of the cell-to-cell variability across conditions could be attributed to cell cycle differences (Fig. 5b). Indeed, with the scRNA-seq data we could identify distinct subpopulations of cycling and non-cycling cells, and further distinguish G2/M and G1/S cells within the cycling cluster (Fig. 5c). Both acute and chronic CENP-A overexpression in the p53-WT cells caused a clear shift in the proportion of cycling to non-cycling cells, a shift that was substantially reduced in the p53-DN cells (Fig. 5d, e). We confirmed these findings by a direct classical analysis of cell cycle using propidium iodide FACS (Supplementary Fig. 4D). Interestingly, we found that the non-cycling populations in the scRNA-seq experiments displayed a striking downregulation of the genes corresponding to cluster 8 in the bulk RNA-seq analysis (Supplementary Fig. 4C), the subset of genes identified as critical for the radiosensitivity phenotype. Given the well-documented role of p53 in senescence^52,53^, we also confirmed that the non-cycling scRNA-seq cluster was consistent with a senescence gene expression signature (Fig. 5f). We thus tested the impact of p53 status and prolonged CENP-A overexpression on cell senescence using Beta-galactosidase senescence assays (Fig. 5g). p53-WT cells showed on average ~19% senescence after 13 days of chronic CENP-A overexpression, compared to <3% in the non-induced control. Meanwhile, p53-DN cells showed approximately half the levels of senescence (~8% and ~1%, respectively). This is consistent with the non-cycling ratios for p53-WT and p53-DN cells in the scRNA-seq data and shows a significant impact on senescence from p53 status. Taken together, our results reveal that CENP-A overexpression causes a clear shift in cell state, promoting acute cell cycle exit and senescence in p53-WT cells that is severely reduced when p53 is defective.

### CENP-A overexpression promotes epithelial–mesenchymal transition in p53-defective cells

**Fig. 6:**
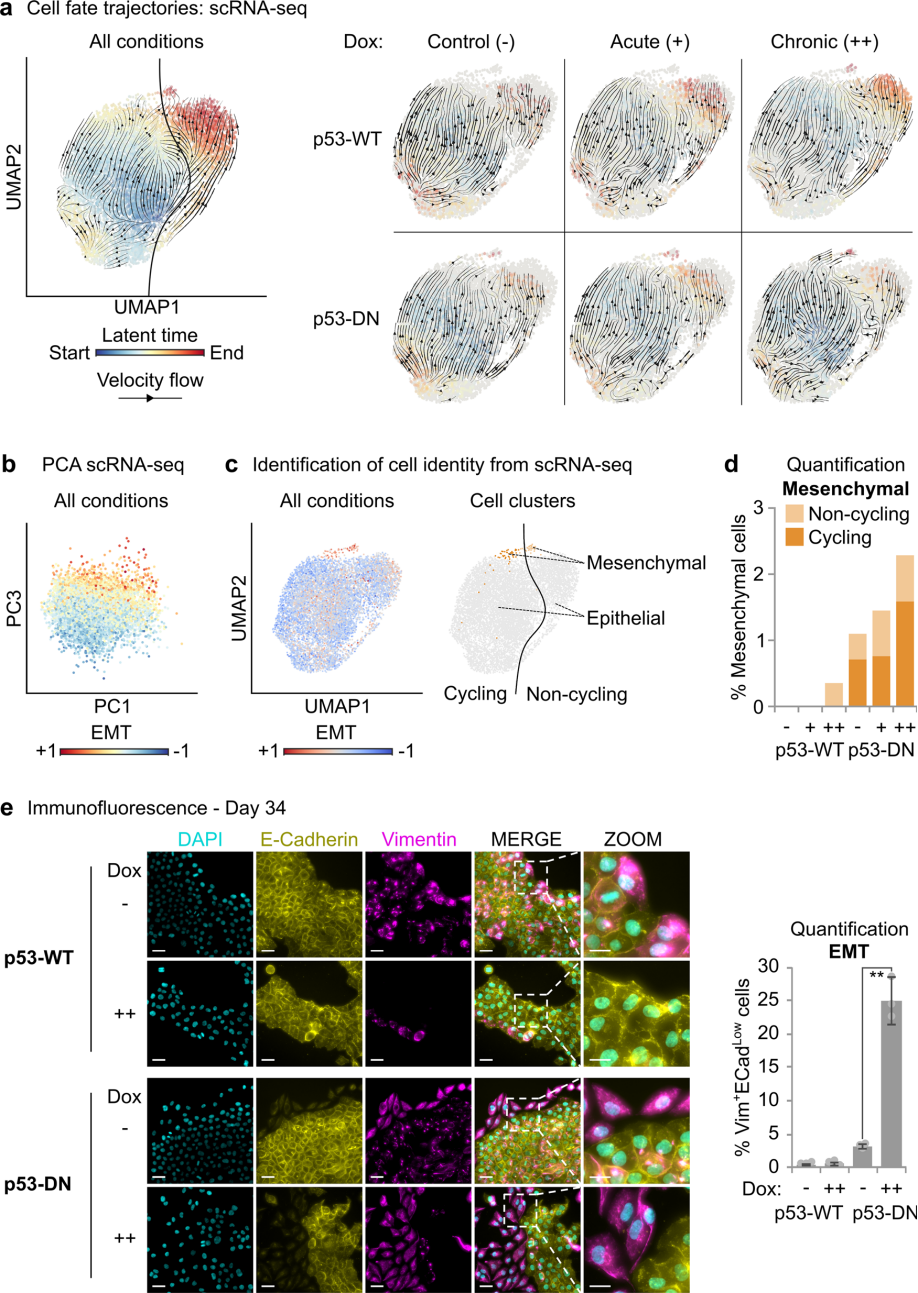
CENP-A overexpression promotes epithelial–mesenchymal transition in p53-defective cells. **a** Cell fate trajectories inferred by RNA velocity analysis of scRNA-seq samples illustrated in Fig. 5a. RNA velocities estimated by dynamical modeling across all samples are depicted in the left panel by a streamline plot (black arrows) showing the main directional flow across cells (gene-averaged velocity vector field in UMAP embedding from Fig. 5). Each cell is colored by the estimated latent time from initial (blue, start) to terminal states (red, end). Left: RNA velocities calculated across all conditions. Right: RNA velocities calculated separately for each condition by stochastic modeling in individual samples. **b** Principal component analysis (PCA) of scRNA-seq experiments. All conditions merged. Each dot represents a single cell on the first (PC1) and third (PC3) principal components, colored by expression of epithelial–mesenchymal transition genes (EMT; Hallmark gene set, MSigDB v6.2). Cell-to-cell variability along the third principal component can be explained by differences in expression of EMT genes. **c** Classification of epithelial and mesenchymal subpopulations based on Leiden clustering after correcting for cell cycle effects. Left: the color gradient shows, for each cell, the relative expression trend of genes involved in EMT (EMT; Hallmark gene set, MSigDB v6.2) in UMAP space. See also Supplementary Fig. 5D for additional EMT signatures. Right: classification of cells as mesenchymal (EMT high cluster, orange) and epithelial (EMT low clusters, gray), divided as either cycling (dark) or non-cycling (pale), as determined in Fig. 5. **d** Stacked bar plot showing the percentage of cells within the mesenchymal cluster per experimental condition. **e** Assessment of EMT by immunofluorescence after 34 days with (++) or without (−) continuous CENP-A overexpression (10 ng/ml Dox). Left: representative max intensity projections: DAPI (cyan), E-cadherin (yellow, epithelial marker), and vimentin (magenta, mesenchymal marker). Scale bars = 40 µm, Zoom = 20 µm. Right: quantification of the frequency of cells with high vimentin surrounding the nucleus and low/absent E-cadherin on the cell membrane for each condition. Plot shows mean and 95% confidence interval from three biological replicates (gray circles). *N* = > 1000 nuclei per condition per replicate. Similar results were obtained in a second independent experiment. ** = two-tailed Welch’s *t* test comparing control p53-DN to p53-DN after chronic CENP-A overexpression; *p* value = 0.0002.

Next, we explored if CENP-A overexpression also impacts cell identity in the different p53 contexts. To do this, we investigated cell fate trajectories across all conditions by estimating transcriptional kinetics based on RNA velocity^54^. In line with Fig. 5, the main trajectory inferred from RNA velocities is the transition of cycling cells to a terminal non-cycling state (see velocity flow and latent time gradient in Fig. 6a). At the same time, we noticed that a minority of cycling cells follows an alternative trajectory converging to a distinct terminal state. This corresponds to a smaller subpopulation comprising both cycling and non-cycling cells. By computing the RNA velocity separately for each condition, we revealed that the main directional flow upon CENP-A overexpression in p53-WT cells is associated with the cell cycle exit trajectory, whereas the alternative cell fate trajectory is mainly followed by p53-DN cells under prolonged CENP-A overexpression. This suggests that CENP-A overexpression in p53-DN cells leads to an alternative cell fate different from cell cycle exit, underlying the emergence of a distinct subpopulation of terminal cells. We thus identified the main clusters of cells after adjusting for cell cycle effects (Supplementary Fig. 5A) and characterized the specific markers associated to each cluster (Supplementary Data 3). The largest clusters corresponded to distinct subpopulations of epithelial cells with differences in expression related to cell metabolism (Supplementary Fig. 5B, C). Interestingly, the alternative cell fate trajectory converged instead to a smaller cluster of cells that were negative for epithelial markers. This cluster showed high expression of mesenchymal genes and broad upregulation of genes involved in EMT (Fig. 6b, c, see also Supplementary Data 3 and Supplementary Fig. 5D). This is consistent with highly advanced EMT or complete mesenchymal transition. The mesenchymal cluster was nearly exclusive to the p53-DN cells (Fig. 6d), consistent with previous studies demonstrating that WT p53 counteracts EMT^55–60^. Remarkably, the highest proportion of mesenchymal cells in the p53-DN condition occurred after chronic CENP-A overexpression, in line with the alternative cell fate trajectory. Given that these cells represent a highly advanced stage of EMT, these findings led us to reexplore our cells for earlier EMT signatures by microscopy. We first noticed that large groups of cells with mesenchymal-like characteristics (reduced cell–cell contacts, distorted cell shape) could be observed in the p53-DN cells by simple brightfield microscopy as early as 10 days after continuous CENP-A overexpression (Supplementary Fig. 6A). At day 34, we immunostained simultaneously for the classic epithelial marker E-cadherin and the mesenchymal marker vimentin (Fig. 6e), to identify cells in a broad range of early to late stages of EMT^61^. The results revealed a major increase in the EMT population after prolonged CENP-A overexpression, which was again nearly exclusive to our p53-DN cells. Indeed, MCF10-2A cells are known to undergo low levels of spontaneous EMT^62^, so it was unclear if this increase in EMT was the result of a stimulation or if CENP-A overexpression—in a p53-defective context—would be sufficient to drive EMT on its own. To assess this, we tested chronic CENP-A overexpression in our p53-defective breast epithelial cancer cell line HCC1954, which does not undergo spontaneous EMT^63^. We did not observe the emergence of EMT in the time frame of our investigation (Supplementary Fig. 6B), suggesting that the major increase in EMT observed in the p53-DN MCF10-2A cells was most likely a stimulation rather than an induction. Together, these assays reveal that chronic CENP-A overexpression promotes a reprogramming of cell identity in p53-defective cells, corresponding to a striking EMT in the p53-DN cell population.

## Discussion

### CENP-A overexpression promotes distinct cell fates depending on p53 status

**Fig. 7:**
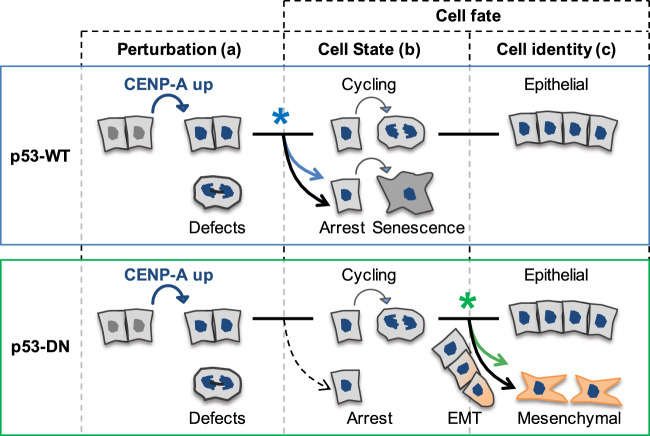
CENP-A overexpression promotes distinct cell fates depending on p53 status. CENP-A overexpression reprograms cell fate with distinct effects on cell state and cell identity that depend on p53 status. Perturbation (**a**) by CENP-A overexpression (in blue) induces mitotic defects in both wild-type p53 (p53-WT, top panel blue) and p53-defective cells (p53-DN, dominant negative, bottom panel green). These defects provoke distinct cell fate decisions according to p53 status, impacting cell state (**b**) or identity (**c**). When p53 is functional, cell state shifts toward acute cell cycle arrest and senescence, reducing self-renewal capacity. Additional stress, like DNA damage from X-irradiation, amplifies this response, resulting in radiosensitivity. Furthermore, functional p53 ensures the preservation of epithelial identity. In contrast, when p53 is defective, the cells evade arrest and continue cycling, allowing CENP-A overexpression to promote epithelial–mesenchymal transition (EMT). * symbol: reprogramming stimulated by CENP-A overexpression.

In this study, we found that CENP-A overexpression promotes radiosensitivity through nongenetic, reversible effects. This is accompanied by major transcriptional reprogramming involving acute cell cycle shutdown and senescence when p53 is active. However, inactivation of p53 suppresses the cell shutdown response, enabling radiotolerance. Strikingly, prolonged CENP-A overexpression also promotes EMT in our p53-DN MCF10-2A cells. Taken together, our findings demonstrate that CENP-A overexpression can promote two distinct cell fates that depend on p53 status: (1) loss of self-renewal capacity and radiosensitivity in p53-WT cells, and (2) reprogramming of cell identity through stimulation of EMT when p53 is defective (Fig. 7). Our work reveals an unanticipated link between a centromeric protein and genome reprogramming with clear implications for tumor evolution. These findings open up exciting avenues for future research and have broad implications for cancer treatment.

### Induced CENP-A overexpression alters cell state and global transcription: implications for cancer treatment

Our work shows that at the timescale of one cell division or less, induced CENP-A overexpression in MCF10-2A cells results in major transcriptional reprogramming across the genome. Switching p53 status from WT to defective counteracted a subset of these transcriptional changes, corresponding to cell cycle genes. But, while our scRNA-seq analyses revealed that these effects on cell cycle genes at the population level can be explained by changes in the proportion of arresting cells, how CENP-A overexpression leads to such broad and rapid changes to gene expression across the genome remains an open question. Given that CENP-A overexpression results in its mislocalization and incorporation into chromatin across the chromosome arms, including numerous genic loci^46,47,50,64^, we envisage two main scenarios for how CENP-A impacts global transcription: (1) the changes reflect a response to defects induced by CENP-A overexpression and/or, (2) CENP-A incorporation into chromatin directly affects transcription at the sites where it is mislocalized. The idea that ectopic CENP-A could directly affect transcription at genic loci has recently been proposed^36^, but remains to be formally assessed. Understanding the underlying mechanisms of these effects could provide important insights into alternative functions of CENP-A and its impact beyond the centromere. On a longer timescale, our scRNA-seq results revealed that prolonged CENP-A overexpression promotes chronic cell cycle arrest in a major proportion of p53-WT cells. Thus, our findings support a model where CENP-A overexpression induces mitotic defects and increases CIN^30^, which, in turn, lead to the activation of p53 and senescence^65,66^. In this way, CENP-A overexpression causes a p53-dependent loss of self-renewal capacity. Additional cell stress from DNA damaging agents, like X-irradiation, would add to the mitotic stress associated with CENP-A overexpression. This would then amplify the p53-dependent shutdown of self-renewal, promoting radiosensitivity. Thus, our findings suggest that differences in p53 status may be able to explain why high CENP-A levels are associated with both sensitivity^34,37,39^ and resistance^31^ to cancer treatments in different patient cohorts. This could have important implications for patient prognosis and treatment strategies. Radiosensitivity associated with high CENP-A levels in p53-WT cancers could represent an opportunity to stratify patients or minimize radiotherapy dose for equal therapeutic outcome with reduced side effects. Furthermore, alternative treatment options for patients with nonfunctional p53 could also be considered, as drugs combatting mutant p53 (including LOF mutations) are currently in development^67,68^. A number of these drugs are now in clinical trials, including direct reactivators of mutant p53 (e.g., PRIMA-1^MET^/APR-246) and drugs designed to return functionality to WT p53 by inhibiting its upstream regulators (e.g., MDM2/X inhibitors)^69^. Here, we showed that transiently reactivating p53 in CENP-A overexpressing HeLa cells—by a transient, clinically relevant, heat shock—was able to significantly increase sensitivity to X-irradiation. Thus, by returning functional p53 status to tumors with high CENP-A, not only could this induce the anti-tumoral effects of activating p53 alone, but it may also promote radiosensitivity due to the effects of CENP-A overexpression on cell state.

### Prolonged CENP-A overexpression and cell identity: implications for stimulating EMT

One of the most surprising findings from our study was the striking stimulation of EMT in p53-DN MCF10-2A cells after prolonged CENP-A overexpression. EMT is a multistage process where epithelial cells, characterized by strong cell–cell junctions and apical–basal polarity, undergo a series of changes to gene expression and morphology to gain a mesenchymal phenotype, including a reorganized cytoskeleton, altered cell shape, and increased cell motility^70^. These changes are highly context dependent, varying substantially among epithelial cells and depending on the factor driving the EMT response^71^. Indeed, the transition to a mesenchymal identity is an essential process during mammalian development, but also occurs aberrantly in epithelial cancers. This can lead to the development of invasive and metastatic properties, with impacts on proliferation, cell plasticity, stemness, and therapeutic resistance^72,73^. In this context, different EMT responses can be driven by distinct signaling pathways, including TGF-β, hypoxia, WNT, and others^74^. In MCF10-2A cells, that spontaneously undergo low levels of EMT^62^, we found that CENP-A overexpression strongly favors the EMT response as an alternative fate to cell cycle exit when p53 is inactivated. However, CENP-A overexpression alone was not sufficient to initiate EMT in the p53-defective HCC1954 breast cancer cells, where EMT does not occur spontaneously but can be induced by TGF-β^63^. Investigating whether high levels of CENP-A favor a mesenchymal fate when the EMT response is induced by different pathways is an important avenue of investigation with critical implications for cancer care. Indeed, CENP-A overexpression has previously been linked to invasion and metastasis in human patients^31,33,34,36,37,39^, but whether this could involve the stimulation of EMT was not known. Thus, the link with EMT provides a mechanism by which CENP-A overexpression could promote these outcomes in patient tumors that lack functional p53. Evidence linking EMT to the direct inhibition of senescence^75,76^, promotion of radioresistance^77–79^, and cancer stemness^80^ imply that EMT may also play an important role in the evasion of senescence, radiotolerance, and increased clonogenic capacity that we observed in our p53-defective cells. Interestingly, the effect of CENP-A overexpression on EMT also suggests that high CENP-A levels could promote stemness, depending on p53 status. Indeed, human pluripotent stem cells naturally overexpress CENP-A^81,82^, and maintain p53 in an inactive state through post-translational regulation^83^. Intriguingly, recent work in Drosophila showed that intestinal stem cells preferentially retain preexisting CENP-A during asymmetric divisions^84^, suggesting that CENP-A nucleosomes may epigenetically mark stem cell identity in this system. Furthermore, Drosophila germ line stem cells showed asymmetric distribution of CENP-A during differentiation and CENP-A overexpression could promote self-renewal^85^. Thus, in addition to its effect on EMT, how CENP-A overexpression contributes to stem cell renewal and pluripotency in human cells will be an important avenue for future research. Importantly, as with the effect of CENP-A overexpression on global transcription, which underlying mechanism is at play remains to be deciphered. Since the initiation of EMT is associated with several of the cell stresses that result from mitotic defects, including genotoxic stress^86^, replication stress^87^, and metabolic stress^88^, among others^89–92^, they are likely important in the process. Interestingly, a recent report by Gomes et al.^93^ demonstrated that the perturbation of the histone H3 variants, H3.1/2 and H3.3, promotes or represses EMT. In particular, knockdown of the H3.1/2-dedicated chaperone CAF-1 caused widespread opening of chromatin, with increased incorporation of H3.3 at the promoters of EMT-inducing transcription factors (e.g., *ZEB1*, *SNAI1*, and *SOX9*), which induced EMT and increased cell migration and invasion. Previously, we found that overexpressed CENP-A hijacks the H3.3-dedicated chaperone Daxx^46^. This results in the mis-incorporation of CENP-A-containing nucleosomes into regions of high histone turnover normally enriched for H3.3. Thus, CENP-A overexpression could have a direct impact on EMT, and transcription in general, through its perturbation of other H3 variants and their dedicated histone chaperones. Deciphering the mechanisms that link CENP-A, EMT, and possibly stemness, will expand our understanding of the direct and indirect molecular consequences of CENP-A overexpression and its impact on tumor evolution.

In conclusion, the interplay of CENP-A overexpression and p53 status alters cell fate, with distinct implications for the role of CENP-A in therapeutic sensitivity, resistance and metastasis.

## Methods

### Cell lines

See Table 1 for cell lines used. Lenti-X 293T cells (632180) were purchased from Clontech. All other parental human cell lines originally from ATCC: HeLa S3 (CCL-2.2), MCF10-2A (CRL-10781), T47D (HTB-133), HCC1954 (CRL-2338). Inducible CENP-A overexpression DLD1 cells obtained from Daniele Fachinetti (Institut Curie). HeLa S3 and MCF10-2A cell lines were authenticated by STR profiling (Powerplex 16 HS). The other cell lines were not authenticated. All cell lines tested negative for mycoplasma contamination. No commonly misidentified cell lines were used in this study.

### Cell culture and CENP-A overexpression

HCT116, HeLa S3, and DLD1 FRT Fbox (+OSTR1-Myc9)+CENP-A-YFP-AID were cultured with DMEM 10% fetal cow serum (FCS) Pen/Strep 1%; MCF10-2A, 1:1 DMEM:Ham’s F12, 20 ng/ml epidermal growth factor, 100 ng/ml cholera toxin, 0.01 mg/ml insulin and 500 ng/ml hydrocortisone, 5% horse serum; HCC1954, RPMI 10% FCS Pen/Strep 1%; Base media from ThermoFisher Scientific. All cell lines tested and confirmed to be mycoplasma free using the Mycoplasma PCR ELISA kit (Sigma). CENP-A overexpression was induced by the addition of Dox to typical growth media at 10 ng/ml (considered 1X), unless otherwise indicated. To maintain overexpression, media and Dox were replaced every 1–3 days. When applicable, the media in the non-induced control was replaced in parallel without Dox. For reversal of CENP-A overexpression, we removed media containing Dox and washed two times with warm PBS then replaced the media without Dox.

### Generation of *TetOn-CENPA-FLAG-HA* cell lines

To obtain an inducible CENP-A overexpression system, we sub-cloned *CENPA-FLAG-HA* from the eCENP-A plasmid^46^ by PCR (forward primer containing EcoRI site: *CACAGTTAGAATTC****ATG****GGCCCGCGCCG*; reverse primer containing AgeI site: *ATCGAATCACCGGT****CTA****GGCGTAGTCGGGCACGT*) and inserted it into the multiple cloning site of the pLVX-TetOne (ClonTech) plasmid by restriction enzyme digestion with EcoRI and AgeI, followed by ligation. To confirm proper integration and lack of mutation in the *CENPA* gene, we then sequenced the *CENPA-FLAG-HA* pLVX-TetOne plasmid around the insertion site. To encapsulate the construct for transduction, we co-transfected Lenti-X 293T cells (ClonTech) with 10 µg *CENPA-FLAG-HA* pLVX-TetOne and packaging vectors 7.5 µg psPAX2 and 2.5 µg pMD2.G using PolyPlus JetPrime transfection reagent according to manufacturer instructions. Media was replaced 4 h after transfection. For infection and stable integration of the construct without selection, we filtered (0.45 µm) supernatant from the transfected cell flask at 24 and 48 h post transfection, added 8 µg/ml of polybrene to the filtered virus-containing media, and added the media directly to the target cell lines. We removed the virus-containing media after 48 h and grew the cells for at least three passages without virus. Then we tested the polyclonal cell lines for the induction of *CENPA-FLAG-HA* by western and immunofluorescence (IF) of CENP-A and HA following 24 h of treatment with 80 ng/ml of doxycycline. We isolated clonal cell lines by serial dilution to single cells. Then tested 10–20 colonies arising from single cells for *CENPA-FLAG-HA* induction, as before. We selected cell lines for further experimentation if they showed a clear homogenous CENP-A and HA increase by IF in ~100% of cells after doxycycline treatment with no detectable background of HA signal by western or IF when no doxycycline was added.

### Change of p53 status

For the p53-WT and p53-DN MCF10-2A cells, we transduced empty vector pWZL Hygro (Scott Lowe, Addgene plasmid #18750) or vector containing the DN *TP53* construct (pBABE-hygro p53DD, Bob Weinberg, Addgene plasmid #9058) into the clonal MCF10-2A *TetOn-CENPA-FLAG-HA* cell line by lentiviral transduction, as above. Cells were selected with hygromycin for >14 days and then passaged in typical media. For heat shock of HeLa S3 *TetOn-CENPA-FLAG-HA cells*, we performed a 1 h 42 °C heat shock on non-induced HeLa *TetOn-CENPA-FLAG-HA* cells and examined total cell extracts by western blotting at various time points following heat shock. Two parallel sets of cells were grown under normal conditions at 37 °C. One set was moved to a 42 °C incubator, while the other remained at 37 °C as a control. After 1 h, we harvested cells for total cell extraction from each condition (time 0 h), then returned both sets to 37 °C. We then harvested cells for total cell extraction at the indicated times after heat shock (1–4 or 6 h: time after HS).

### Total cell extracts (TCEs)

We harvested cells by trypsin, counted cells by Beckman Automated Cell Counter, and spun down at 300 g, 5 min, washed 1X in PBS, spun again, aspirated PBS, and froze pellets at −20 °C. Cell pellets were incubated for 15 min at room temperature with 300 µl per 1 million cells of 1.2X LDS Sample Buffer (NuPAGE) containing 1.2X Sample Reducing Agent (NuPAGE) and 125 kU/ml Pierce Universal Nuclease Buffer for Cell Lysis, then heated at 95 °C for 10 min, vortexed, spun down, and cooled briefly on ice prior to western blotting.

### Western blotting

Total cell extracts were loaded at one to four different concentrations, as indicated, onto premade NuPAGE Bis-Tris 4–12% gradient gels in an XCell 4 Sure-Lock SDS-PAGE chamber with 1X NuPAGE MES SDS Running Buffer, with a PageRuler (ThermoFisher Scientific) molecular weight marker. Gels were run at 130–150 v for 1 h to 1 h15 min. We transferred protein to a 0.2 µm nitrocellulose membrane by BioRad Trans-Blot Turbo, mixed-molecular weight setting, or semi-dry transfer (BioRad, 15 v, 1.5 h). Membranes were stained with Pierce reversible protein stain to detect bulk protein and assess quality of transfer, then cut, blocked in 5% milk-PBST (0.1% Tween-20) for 1 h, RT, and incubated overnight with rocking at 4 °C with primary antibodies in 5% milk-PBST containing 0.02% sodium azide. Membranes were washed 3X 10 min in PBST, then incubated 1 h (RT) with horseradish peroxidase secondary antibodies in 5% milk-PBST, washed 3 × 10 min in PBST and exposed with SuperSignal West Pico Chemiluminescent Reagent or, for high sensitivity exposures, SuperSignal West Femto Maximum Sensitivity Reagent, and imaged using a ChemiDoc Touch system and ImageLab software. See Supplementary Figs. 7–10 for all uncropped blots. Primary antibodies: CENP-A 1:500 (2186 Cell Signaling Technology (CST)), H4-pan 1:2500 (05-858, Sigma-Aldrich), HJURP 1:300 (HPA008436 Sigma-Aldrich), γTubulin 1:10000 (T5326 Sigma-Aldrich), p53 (1C12) 1:1000 (2524 CST), p21 1:500 (556431 BD Pharmingen), phospho-p53 (Ser15) 1:500 (9284 CST). Secondary antibodies:Jackson ImmunoResearch, 1:10000, donkey anti-mouse or donkey anti-rabbit.

### Immunofluorescence (IF)

Glass coverslips were coated with collagen (1 µg/ml) + fibronectin (1 µg/ml) in PBS, 30 min, RT, washed in PBS and added to culture dishes prior to cell seeding. After at least 24 h of growth, coverslips were washed 3X with PBS, fixed with 2% PFA, 20 min, RT, washed 3X PBS, incubated 5 min with 0.3% Triton-X 100 in PBS, washed 3X PBS, blocked in filter-sterilized 5% BSA-PBST (0.1% Tween-20) for 20–30 min, RT, and incubated 1–1.5 h, RT, with primary antibodies in 5% BSA-PBST. Coverslips were then washed 3X 5 min in 5% BSA-PBST, and incubated 30 min with secondary antibody in 5% BSA-PBST, RT, in darkness. DAPI was added directly to secondary antibody solution (final concentration 1/4000) and incubated in darkness, RT, 5 min, followed by 3X PBS wash. Coverslips were inverted onto microscope slides with ~10 µl of VectaShield and imaged with a Zeiss Axiovert Z1 microscope or Inverted Widefield Deltavision Core Microscope (Applied Precision) with CoolSNAP HQ2 camera, using MetaMorph (Zeiss microscope) or Softworx (Deltavision microscope) software. Sample sizes for microscopy quantifications were determined according to the number of countable cells within at least three different fields per replicate. More fields were imaged if fewer cells were present than average. Image analysis performed with ImageJ software. Primary antibodies: CENP-A (3-19) 1:300 (ADI-KAM-CC006-E Enzo Life Sciences), E-cadherin (24E10) 1:200 (3195S CST), γH2AX 1:250 (2577 CST), vimentin (N-term) 1:100 (5741S Progen). Secondary antibodies: 1:1000 Alexa Fluor donkey anti-mouse IgG (H + L) 488, 1:1000 Alexa Fluor goat anti-rabbit IgG (H + L) 488 or 594, 1:1000 Alexa Fluor goat anti-Guinea Pig IgG (H + L) 594.

### Colony formation assays (CFAs)

At day −1, cells are trypsinized and diluted to single-cell level (300–600 cells per well, in triplicate) in six-well plates. At day 0, one set of cells is irradiated by CIXD Dual Irradiator or Philips X-ray tube X-ray generator (4 Gy, unless otherwise indicated), while a control set remains unirradiated (0 Gy). When the cells have had sufficient time to form visible colonies, depending on their typical speed of growth (7–14 days), they are washed gently with PBS, stained with 1% Crystal Violet 20% Ethanol solution for 15 min, washed with water, allowed to dry, scanned and counted by eye from the acquired images with the counter blinded to the conditions. Unless otherwise indicated, ratio of surviving colonies is calculated as the number of colonies formed after 4 Gy of X-irradiation divided by the number of colonies formed when not irradiated. Cells are considered sensitized to irradiation by CENP-A overexpression when the CENP-A overexpression condition has a significantly lower survival ratio compared to the non-induced control. Cells are considered tolerant to CENP-A overexpression if the survival ratio is not significantly affected by the CENP-A overexpression. See Supplementary Data 1 for all counts and calculations.

### Bulk RNA-seq

Sample preparation: we grew MCF10-2A *TetOn-CENPA-FLAG-HA* cells expressing either empty vector (p53-WT) or dominant-negative p53 (p53-DN) with 0X Dox (no Dox), 1X Dox (10 ng/ml), or 10X Dox (100 ng/m) for 24 h. At time 0, we irradiated one set of cells by X-ray generator (4 Gy), while a control set remained unirradiated (0 Gy). Six hours later, we extracted RNA for RNA-seq. All conditions tested in duplicate. We extracted RNA directly from culture dishes using the Qiagen RNeasy Mini kit according to the manufacturer’s instructions and confirmed the quality and quantity of the RNA by TapeStation and NanoDrop. mRNA library preparation and sequencing were performed by the next-generation sequencing (NGS) platform, Institut Curie, using the Illumina TruSeq Stranded mRNA kit and NovaSeq 6000 sequencer. Library preparation and sequencing of irradiated and nonirradiated samples were performed on different dates, with a single repeat of one sample included for batch correction.

### Bulk RNA-seq analysis

Reads were aligned to the human reference genome (GRCh38 assembly) based on Ensembl gene annotation (release 95) with hisat2 (version 2.1.0^94^), run in paired-end mode with default parameters. Gene-level counts were computed from primary alignments with MAPQ > 2 using featureCounts (Subread package version 1.6.3^95^) in paired-end mode with the -s 2 option for reverse-stranded libraries. Raw counts were normalized for differences in library size (counts per million) and across samples (via trimmed mean of *M* values normalization, TMM) using edgeR (version 3.28.0^96^). Differential expression analyses were performed with edgeR^97^. We assessed the stand-alone and combined response to each treatment (CENP-A overexpression at increasing Dox concentrations, p53-DN, and X-irradiation) by fitting a quasi-likelihood negative binomial generalized log-linear model (GLM), including both additive and synergistic effects for all treatment combinations. A batch coefficient was also included to account for potential batch effects. Full details about the variable encoding and GLM formulation are provided in Supplementary Data 2 (sheet 1). The effect of each treatment, both stand-alone and combined, was evaluated via quasi-likelihood *F*-test on the respective GLM coefficient, followed by multiple testing correction via the Benjamini–Hochberg method (Supplementary Data 2, sheet 2). A false discovery rate (FDR) cut-off of 0.05 was used to identify DEGs. For proportional Venn diagrams, we only considered DEGs that change with CENP-A overexpression, p53 status or X-irradiation, since we could not detect significant interaction effects for most treatment combinations (Supplementary Data 2, sheet 2). Mean-centered TMM-normalized counts were used for PCA and hierarchical clustering analyses via Ward’s variance minimization method (Supplementary Data 2, sheets 3 and 4). For hierarchical clustering, we tested the combined effect of all coefficients, excluding batch, and included any DEG showing significant differences relative to baseline at 0.05 FDR. Functional enrichment analyses for KEGG pathways were carried out using the WebGestaltR package (version 0.4.2^98^). GSEA for CENP-A effects were performed on all expressed genes ranked by fold change and *p* value upon overexpression, i.e., log_2_ fold change *−log_10_ *p* value of the Dox coefficient (Supplementary Data 2, sheets 5 and 6). Pathways associated to specific gene clusters were identified by ORA after hierarchical clustering of all DEGs via Ward’s method (Supplementary Data 2, sheets 7 and 8). Coordinated changes in expression within DEG clusters were evaluated by assessing the distribution of mean-centered expression values (averaged per condition) for all genes in a given cluster. For genes in cluster 8, we fit a linear mixed model (LMM) to estimate the fixed effects of p53 inactivation and X-irradiation at each level of Dox (relative to nonirradiated p53-WT samples), with a varying intercept for each gene. We similarly estimated the effect of CENP-A overexpression at increasing levels of Dox by fitting a separate LMM across all conditions. The LMM formulation, model estimates, and pairwise comparisons by *F*-test are reported in Supplementary Data 2 (sheets 9 and 10), along with adjusted *p* values after Bonferroni correction. Bulk RNA-seq analyses were carried out with custom Python scripts. pandas (version 0.24.2), NumPy (version 1.16.2), SciPy (version 1.3.1), statsmodels (version 0.10.1) and scikit-learn (version 0.21.3) libraries were used for data manipulation, statistical analysis, and unsupervised learning. matplotlib (version 2.2.4), matplotlib-venn (version 0.11.5), and seaborn (version 0.9.0) were used for plotting and statistical data visualization. R packages were imported into Python using rpy2 (version 2.8.4).

### Reverse transcription quantitative PCR (RT-qPCR)

We cultured two replicates for each cell type and condition in separate 60 mm dishes for 1 day in typical media, then added 0, 10, or 100 ng/ml Dox for 24 h. We extracted RNA directly from plates using the Qiagen RNeasy Mini kit with in-column DNase I treatment, according to the manufacturer’s instructions. RNA concentrations were quantified by NanoDrop and diluted to 125 ng/µl and confirmed by NanoDrop. To obtain cDNA, we then performed reverse transcription PCR on 1000 ng of RNA for each sample using the SuperScript III First-Strand Synthesis SuperMix kit, according to the manufacturer’s instructions. We performed quantitative PCR using Power SYBR Green qPCR master mix with three technical replicates in 384-well plates using the QuantStudio 5 Real-Time PCR System. Relative expression was calculated subtracting Ct for each gene of interest from the Ct for *PPIA* for each technical replicate (ΔCt) and took the mean ΔCt. For normalization, we then subtracted the mean across biological replicates of the p53-WT 0X condition for each gene of interest (ΔΔCt). Relative expression was then calculated as the fold change of ΔΔCt (2^ΔΔCt^) for each biological replicate. See Supplementary Data 1 for Cts and calculations. Primers used were as follows (desalt): CDC20 F e8/9 *GGGCTGTCAAGGCCGTAG*, CDC20 R e10 *GACCAGAGGATGGAGCACAC*; MCM2 F e8/9 *TTGACAAGATGAATGACCAGGAC*, MCM2 R e10 *GAGAAAGTCAGCGAGGGGTC*; AURKB F e1/2 *TTGGACCCCAGCTCTCCTC*, AURKB R e3 *GACAAGTGCAGATGGGGTGA*; NDC80 F e4 *GTGCCGACAGCTTTGATGAG*, NDC80 R e5/6 *ACGACTCTAGACGATTCGGTTC*; PLK1 F e6/7 *GTCAGGCAAGAGGAGGCTG*, PLK1 R e8 *TCATTGAAGAGCACCCCCAC*.

### Comet assays

Prior to irradiation, cells were harvested and submerged into media containing low-melting agarose on ice. When hardened, cells were exposed to the indicated dose of γ-irradiation (GSR D1 gamma ray). Either immediately, or at the indicated times postirradiation, the cells in the gel were submerged in alkaline solution and run by gel electrophoresis to form comets to visualize the levels of DNA damage. Comet assays were performed by the RadExP platform, Institut Curie, according to the Trevigen Single Cell Electrophoresis Assay alkaline comet protocol. Automatic counting and measuring of comets on Trevigen slides (two wells used for each condition, per experiment), imaged using the Metafer system with the “comet” module from MetaSystems. See Supplementary Data 1 for mean Olive Tail Moment per condition for each experiment and calculations.

### Micronuclei

We seeded cells into dishes containing collagen/fibronectin-coated glass coverslips 48 h prior to fixation, added indicated concentrations of Dox, and allowed the cells to grow for 24 h (day 1) or passed cells and replaced Dox every 2–3 days until day 8. Cells were fixed, prepared for IF, and nuclei were imaged with a Zeiss Z1 inverted microscope using a 10X objective. We counted micronuclei using a custom ImageJ macro with at least three fields and >1000 nuclei counted per condition. See Supplementary Data 1 for all counts and calculations.

### Multicolor fluorescence in situ hybridization (mFISH) karyotyping

Cells were grown with or without 10 ng/ml Dox for 15 days, then treated with colcemid (100 ng/ml, Roche) for 1.5 h and prepared as previously described for mFISH (MetaSystems) staining^99^. Briefly, mitotic cells were collected by mitotic shake-off after a short trypsin treatment and centrifuged at 1000 rpm for 10 min. Cell pellets were resuspended in 75 mM KCl and incubated for 15 min in a 37 °C waterbath. Carnoy fixative solution (methanol/acetic acid, 3:1) was prepared and 1:10 volume added on the cells, before centrifugation at 1000 rpm for 15 min. Cells were then fixed 30 min at room temperature in the Carnoy solution, centrifuged and washed once more with fixative. Minimum volume of fixative was left to resuspend the pellet and cells were dropped onto clean glass slides. mFISH staining was performed following manufacturer’s instructions (MetaSystems). The Metafer imaging platform (MetaSystems) and the Isis software were used for automated acquisition of the chromosome spread and mFISH image analysis. Chromosome rearrangements and specific chromosome counts for each spread were assessed and counted by eye from the automated chromosome spread images, with the researchers blinded to the conditions. Losses and gains of chromosomes per cell were calculated as the sum of the absolute difference from the mode for each chromosome of the p53-WT no Dox control. New chromosome rearrangements per cell were determined as the total number of structural chromosomal anomalies observed per spread, excluding the ones that were common to most or all p53-WT no Dox spreads. Potential acrocentric fusions were not considered in our study. See Supplementary Data 1 for all counts and calculations.

### Growth curve/proliferation assays

Cells that had never been exposed to Dox were trypsinized, counted using a Beckman Automated Cell Counter, and seeded into nine culture dishes with equivalent cell numbers (corresponds to MCF10-2A non-inducible control, plus the p53-WT and p53-DN TetOn-CENP-A-FLAG-HA cell lines, at three Dox concentrations each, all grown in parallel). The next day (day 0), Dox was added in triplicate at the indicated concentrations (0, 10, or 100 ng/ml), corresponding to 0X, 1X, or 10X Dox. At day 1, we trypsinized and counted the cells from all conditions, and seeded equivalent numbers of cells into fresh media. We immediately added the corresponding concentrations of Dox to the culture dishes. From then on, every 2–6 days cells were trypsinized, counted, and replated, as plotted, with media containing fresh Dox of corresponding concentrations replaced every 2–3 days. Biological replicates were maintained separately throughout the entirety of the experiment. See Supplementary Data 1 for all counts and calculations.

### Single-cell RNA-seq

Sample preparation: we grew MCF10-2A *TetOn-CENPA-FLAG-HA* cells expressing either empty vector (p53-WT) or dominant-negative p53 (p53-DN) without Dox or with continuous exposure to Dox (10 ng/ml, ++, chronic) for 69 days in parallel. Cells in the no Dox condition were split into two dishes at day 67 and Dox was added to one set on day 68 for 24 h of CENP-A overexpression (10 ng/ml, +, acute), while the other remained without Dox (−, control). We prepared samples in singlet according to the 10x Genomics Sample Preparation Demonstrated Protocol. In brief, we harvested cells by trypsinization, resuspended in typical media and mixed thoroughly by pipette, passed cells through a 40 µm cell strainer, and counted cells by Beckman Automated Cell Counter. We resuspended cells in 1x PBS + 0.04% BSA, mixed, centrifuged gently, aspired supernatant, and repeated wash two times. We passed cells through another cell strainer (40 µm), counted again and diluted to a final concentration between 700 and 1200 cells/µl. We then proceeded directly to GEM generation and barcoding at the NGS platform, Institut Curie, using the Single-cell 3′ Reagent Kits v2 protocol with a targeted cell recovery of 2000 cells per sample, followed by post GEM-RT cleanup and cDNA amplification, then 3′ Gene Expression Library Construction, according to the manufacturer’s instructions. Sequencing was performed by the NGS platform with a NovaSeq 6000 sequencer.

### Single-cell RNA-seq analysis

Reads were pseudoaligned to Ensembl transcripts (GRCh38, release 95) using kallisto (version 0.46.0^100^) with the -x 10 × 2 option for Chromium Single Cell 3′ v2 chemistry. Count matrices were generated from sorted BUS files using bustools (version 0.39.2^101^), after barcode correction with the 10x v2 whitelist. For each sample, we selected cells via distance-based estimation of the knee in the cumulative distribution of distinct UMIs per barcode (unique molecular identifiers) using UMI-tools (version 1.0.0^102^). Sample matrices were thus converted to AnnData objects and concatenated for further quality control (QC) and analysis with Scanpy (version 1.4.6^103^). From the merged count matrix, we filtered out cells with abnormal levels of mitochondrial RNA, after adjusting for depth and total number of detected genes. Outliers were detected by covariance estimation (elliptic envelope with 5% contamination) using the number of genes, total counts (log-transformed), and mitochondrial counts (log-transformed) as features for outlier detection. Genes detected in <100 cells (after filtering) were also excluded. For comparison of scRNA-seq and bulk RNA-seq results, raw counts from matched samples were pooled across all cells, TMM normalized and mean centered.

For single-cell analyses, raw counts were normalized per cell, log-transformed, and adjusted for differences in sequencing depth via linear regression (using log-transformed total counts per cell). PCA was performed on the top 3000 highly variable genes (HVGs) after scaling. We computed a neighborhood graph using the first 20 principal components, adjusting for technical differences by batch alignment with BBKNN (version 1.3.9^104^). Uniform Manifold Approximation and Projection (UMAP) was used to visualize cell-to-cell variation in a low-dimensional (2D) space. Cell subpopulations were detected by Leiden clustering (at 0.5 resolution). Epithelial and mesenchymal cells were separately identified with the same procedure, but normalized counts were also adjusted for cell cycle differences (using the G1/S and G2/M signature) prior to scaling and PCA. For each cluster, genes were ranked by one-vs-all logistic regression to identify the top markers (see Supplementary Data 3). Boundaries among clusters were computed via Support Vector Classification with a third degree polynomial kernel. Gene sets for computing expression signatures were retrieved from Cyclebase v3.0 (cell cycle, G1/S and G2/M)^105^ or MSigDB v6.2 (hallmark and curated gene sets related to senescence, EMT, and cell metabolism)^106^. References for each of the signatures are provided in Supplementary Data 3 (sheet 1). The scores (color gradients) are a measure of the average expression of the given set of genes, relative to a set of randomly sampled genes at comparable expression levels. Log-transformed normalized counts (prior to scaling) were used for the calculation. The score is then rescaled across cells from −1 (lowest) to +1 (highest).

For RNA velocity analyses, an additional index including both cDNA and intron sequences was generated from Ensembl annotations (GRCh38, release 95) using the kb-python wrapper (version 0.24.4) for pseudoalignment and quantification with kallisto and bustools (La Manno workflow). Filtered count matrices for both spliced and unspliced transcripts were added to the preprocessed data and analyzed using the scVelo package (version 0.2.2). RNA velocity was first evaluated across all samples for the top 3000 HVGs by fitting a dynamical model estimating the kinetic rate parameters for each gene, as well as the latent time underlying the overall kinetics^54^. To check whether the velocity flow changes by condition, we further estimated RNA velocities in individual samples by stochastic modeling. The trajectories across all conditions, and for each sample, were visualized in the precomputed UMAP embedding using a streamline plot that shows the directional flow across cells (considering velocities with a minimum mass of 3 and applying a smoothing factor of 0.8). In all UMAP plots, the latent time is represented by a color gradient ranging from 0 (initial state, blue) to 1 (terminal state, red).

Singe-cell RNA-seq analyses were carried out with custom Python scripts using Scanpy (version 1.4.6), scVelo (version 0.2.2), BBKNN (version 1.3.9), UMI-tools (version 1.0.0), pandas (version 0.25.0), NumPy (version 1.17.0), scikit-learn (version 0.21.3), matplotlib (version 3.3.0), and seaborn (version 0.9.0).

### Beta-galactosidase senescence assay

We tested samples in singlet and duplicate for senescence using the Cell Signaling Technologies Senescence β-Galactosidase Staining Kit, according to the manufacturer’s instructions. After 24 h with the stain, we imaged at least three fields per sample by brightfield microscopy using a 10X objective on a Nikon Eclipse TS100 microscope. Cells were counted manually from the acquired images with the counter blinded to the conditions. Only cells with dark blue staining were considered senescent. See Supplementary Data 1 for all counts and calculations.

### Propidium iodide FACS

To analyze cell cycle changes by FACS, we harvested cells with trypsin and fixed with cold 70% ethanol. We stained fixed cells with propidium iodide using FxCycle PI/RNase solution (ThermoFisher Scientific), according to the manufacturer’s instructions. FACS was performed on three biological replicates with a FACS Accuri 6 machine and cell cycle was assessed on a minimum 15,000 gated cells, excluding cell debris and doublets, with FlowJo software (V10.1r5). See Supplementary Fig. 4D for example of gating strategy and Supplementary Data 1 for all counts and calculations.

### Statistics and reproducibility

Specific statistical tests, sample sizes, number of replicates, and information on the reproducibility of experiments are indicated in the figure legends and/or methods for each experiment. See Supplementary Data 1 for all source data pertaining to main figures and supplementary figures, including specific *N* values and *p* values from statistical tests. Biological replicates are defined as different sets of cells cultured under the same experimental conditions, processed separately but in parallel (at the same time). Independent experiments are defined as different sets of cells cultured under the same experimental conditions that were cultured (and processed) at different times. No statistics were derived from technical replicates (repeats/replicates of processed samples). No data were excluded from the analyses. For QCof scRNA-seq data, cells with abnormal levels of mitochondrial RNA, after adjusting for depth and total number of detected genes, were filtered out via outlier detection. Outliers were detected by covariance estimation (elliptic envelope with 5% contamination) using the number of genes, total counts (log-transformed) and mitochondrial counts (log-transformed) as features for outlier detection. Genes detected in <100 cells (after filtering) were also excluded. All experiments, with the exception of sequencing and mFISH experiments, were repeated independently at least once, as indicated in the figure legends. All repeats were consistent with the data reported. Number of biological replicates tested are also indicated for each experiment in the figure legends: typically 3 or 6 biological replicates were used for CFA experiments, 2 or 3 for IF and senescence quantifications, 1 for western blots, 3 for proliferation assays, and 2 for bulk RNA-seq. Single-cell RNA-seq was performed in singlet, but were validated by comparison to bulk RNA-seq. For all manual quantifications (CFAs, IF, senescence, micronuclei, mFISH), associated images were randomized and de-labeled prior to analysis to aid in blinding the researchers to the experimental conditions.

### Reporting summary

Further information on research design is available in the Nature Research Reporting Summary linked to this article.

## Supplementary information

Supplementary Information

Description of Additional Supplementary Files

Supplementary Data 1

Supplementary Data 2

Supplementary Data 3

Reporting Summary

## Data Availability

Bulk- and single-cell RNA-seq data have been deposited in ArrayExpress under accession numbers E-MTAB-9867 and E-MTAB-9861, respectively. All other relevant data can be found in Supplementary Data 1 or are available from the authors upon request.
