# Supplementary material for: CENP-A overexpression promotes distinct fates in human cells, depending on p53 status
Source: Commun Biol. 2021 Mar 26;4:417. doi: 10.1038/s42003-021-01941-5 (PMC7997993; doi:10.1038/s42003-021-01941-5)
Supplement: Supplementary file 1 — Supplementary Information [file 42003_2021_1941_MOESM1_ESM.pdf]

Supplementary Information

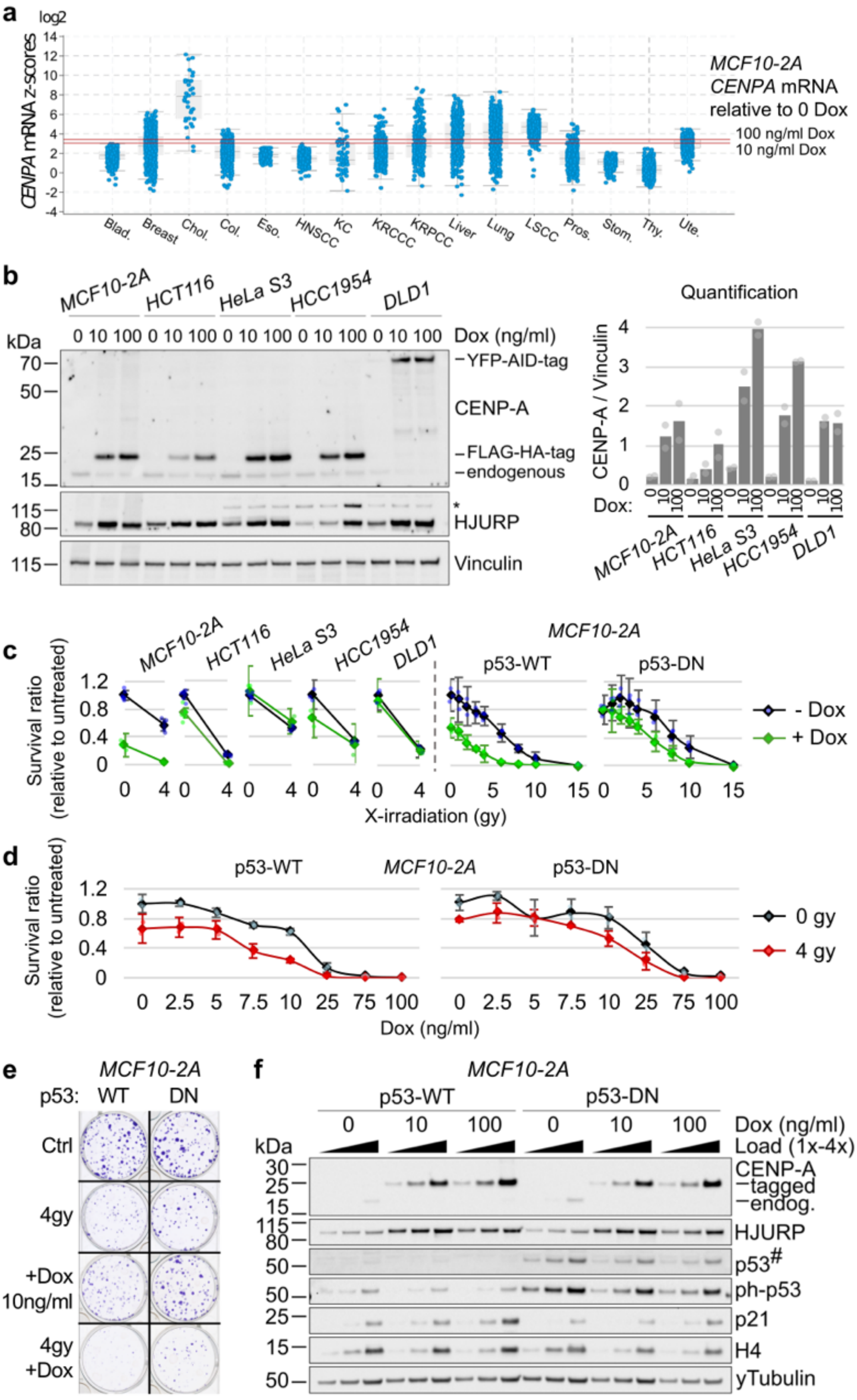

Supplementary Figure 1

Supplementary Figure 1 – Related to Figures 1, 2, 3, 4 and Supplementary Figure 3. Defined range of p53-dependent radiosensitivity in MCF10-2A cells

A) Box and dot plots indicating *CENPA* expression in patient tumours relative to normal samples (z-score, log2 RNA SeqV2 RSEM) from cBioPortal (TCGA PanCancer Atlas studies). The plot was generated using the cBioPortal web interface, and modified with red lines showing approximate *CENPA* levels in our system at increasing doses of doxycycline (Dox) relative to no Dox control (see RNA-seq Figure 4). X-axis indicates study of origin (all TCGA PanCancer Atlas): Bladder Urothelial Carcinoma (Blad.), Breast Invasive Carcinoma (Breast), Cholangiocarcinoma (Chol.), Colorectal Adenocarcinoma (Col.), Esophageal Adenocarcinoma (Eso.), Head and Neck Squamous Cell Carcinoma (HNSCC), Kidney Chromophobe (KC), Kidney Renal Clear Cell Carcinoma (KRCCC), Kidney Renal Papillary Cell Carcinoma (KRPCC), Liver Hepatocellular Carcinoma (Liver), Lung Adenocarcinoma (Lung), Lung Squamous Cell Carcinoma (LSCC), Prostate Adenocarcinoma (Pros.), Stomach Adenocarcinoma (Stom.), Thyroid Carcinoma (Thy.), Uterine Corpus Endometrial Carcinoma (Ute.).

B) Left: Western blot using total cell extracts from indicated cell lines after 24h with 0, 10, or 100 ng/ml of Dox. Primary antibodies indicated on the right. Exogenous CENP-A (tagged) is distinguished from endogenous CENP-A by increased molecular weight. Vinculin used as loading control. * = non-specific band. Right: Quantification of CENP-A Western blot intensity (endogenous + exogenous) relative to vinculin for each condition. Plots show mean (bars) of quantifications from two blots (dots) using ImageLab 6.1 software.

C) Left of dashed line: Line plots of colony formation assay (CFA) results from Figure 2B, except showing survival ratios relative to untreated condition (0 Gy, no Dox) for each cell line. Right of dashed line: CENP-A overexpression causes p53-dependent X-irradiation sensitivity at a defined range of X-irradiation doses. Line plots of CFAs for MCF10-2A *TetOn-CENPA-FLAG-HA* cell lines stably expressing either empty vector or p53-DN were treated with a range of X-irradiation doses (0-15 Gy, X-axis). Plots show mean (diamonds) and 95% confidence interval for one experiment with three or six biological replicates (small circles). Green: CENP-A overexpression (+ Dox (10 ng/ml)). Black: No Dox (- Dox).

D) p53-dependent X-irradiation sensitivity is specific to mid-range doxycycline levels. CFAs as in c (right), except using a range of doxycycline doses (0-100 ng/ml, X-axis), instead of a range of X-irradiation doses. Black: unirradiated (0 Gy). Red: 4 Gy X-irradiation. Plots show mean and 95% confidence interval for survival ratio relative to untreated p53-WT control for one experiment performed in triplicate.

E) Representative images of p53-WT and p53-DN MCF10-2A CFA stains, showing single 3.5cm well of 6-well plate. Conditions indicated on the left. Ctrl: untreated (0 Gy, no Dox).

F) Western blot of MCF10-2A *TetOn-CENPA-FLAG-HA* cells stably expressing either empty vector (p53-WT) or p53-DN. Total cell extracts after 24h with Dox (0, 10, or 100 ng/ml, as indicated). Load: 1x, 2x, 4x, where 1x load = ~13300 cells. Primary antibodies are indicated on the right. [#] = high sensitivity ECL exposure. H4 and γ-tubulin used as loading controls. Note that 100 ng/ml Dox (referred to as 10X in Figure 5) corresponds to the Dox dose that produces the maximum CENP-A overexpression obtainable in our system, corresponding to ~2X more CENP-A protein compared to 10 ng/ml Dox (referred to as 1X in Figure 5).

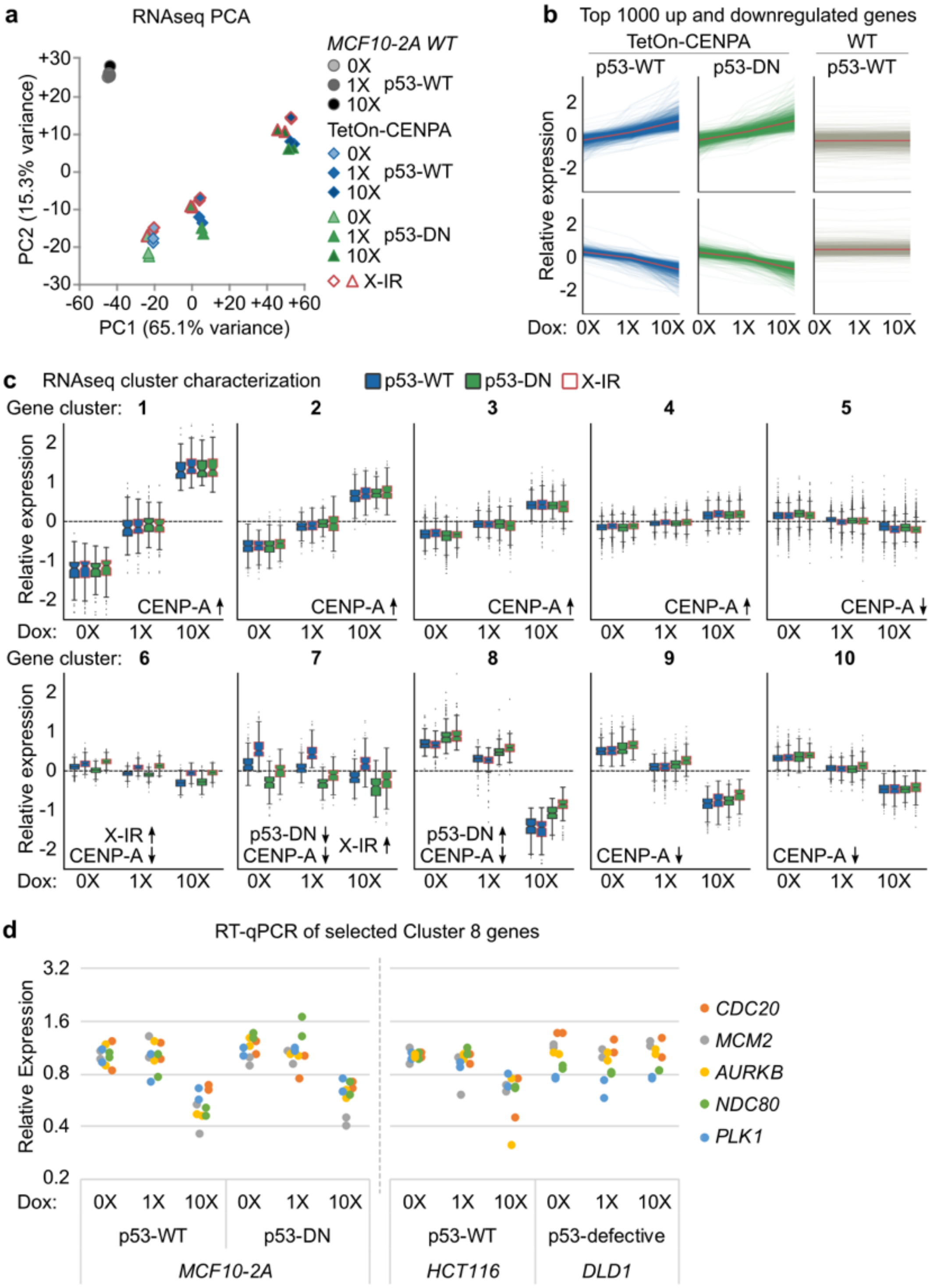

Supplementary Figure 2

Supplementary Figure 2 – Related to Figure 4 and Supplementary Table 1. RNA-seq non-inducible controls and gene cluster characterization

A) Principal component analysis (PCA) of gene expression profiles from bulk RNA-seq data, as described in Figure 4A, but including non-inducible (MCF10-2A WT) controls. Each dot represents a single sample. Legend indicated below. Red outline indicates X-irradiated samples (X-IR, 4 Gy). PC1 on X-axis shows that most of the variance can be attributed to increasing CENP-A levels across inducible cells. Non-inducible controls cluster separately and Dox treatment does not drive major differences in their expression.

B) Effect of Dox on gene expression for inducible and non-inducible MCF10-2A cells. Relative expression from RNA-seq data of the top 1000 upregulated genes (top) and top 1000 downregulated genes (bottom) by CENP-A overexpression in MCF10-2A *TetOn-CENPA-FLAG-HA* cells, plotted by increasing Dox concentrations for each cell line. Each line represents an individual gene, while the average is shown in red for each cell line. These genes are not significantly affected by Dox in the non-inducible control.

C) Gene clusters 1-10 corresponding to heat map in Figure 4D. Box plots showing the distribution of relative expression levels of DEGs in each cluster, averaged by experimental condition. Each data point represents the mean expression level per condition, relative to average, for a particular DEG within the cluster (mean-centered counts, log_2-transformed and TMM-normalized). Center lines show the medians; box limits indicate the 25th and 75th percentiles; whiskers extend 1.5 times the interquartile range from the 25th and 75th percentiles; outliers represented by dots. Main effects of experimental conditions are summarized at the bottom of each plot: up arrow indicates upregulation of genes in the cluster with CENP-A overexpression, X-irradiation, or p53-DN; down arrow indicates downregulation. Clusters 1 to 4 comprise genes that are upregulated as CENP-A is overexpressed, while genes in Clusters 5 to 10 are downregulated, each to varying degrees. Interestingly, genes in Clusters 6 to 8 also showed a coordinated response to radiation treatment or p53 inactivation. Importantly, the only cluster where p53 inactivation showed opposite effects to CENP-A overexpression was Cluster 8. Cluster 8 is also displayed in Figure 4E, duplicated here for direct comparison with other clusters. The top 10 enriched KEGG pathways for each DEG cluster are provided in Table S1.

D) Reverse transcription quantitative PCR (RT-qPCR) for selected Cluster 8 genes, with *PPIA* as a reference gene. RNA extraction, DNase treatment and reverse transcriptase PCR of p53-WT and p53-DN MCF10-2A TetOn-CENPA-FLAG-HA cells, HCT116 TetOn-CENPA-FLAG-HA and DLD1 TetOn-CENPA-YFP-AID cells after 24h with 0, 10, or 100 ng/ml Dox (0X, 1X, 10X, respectively). Plots show gene expression for two biological replicates (dots) relative to *PPIA,* normalized to p53-WT 0X condition (MCF10-2A p53-WT 0X or HCT116 0X)(fold change ΔΔCt) on Log_2 scale.

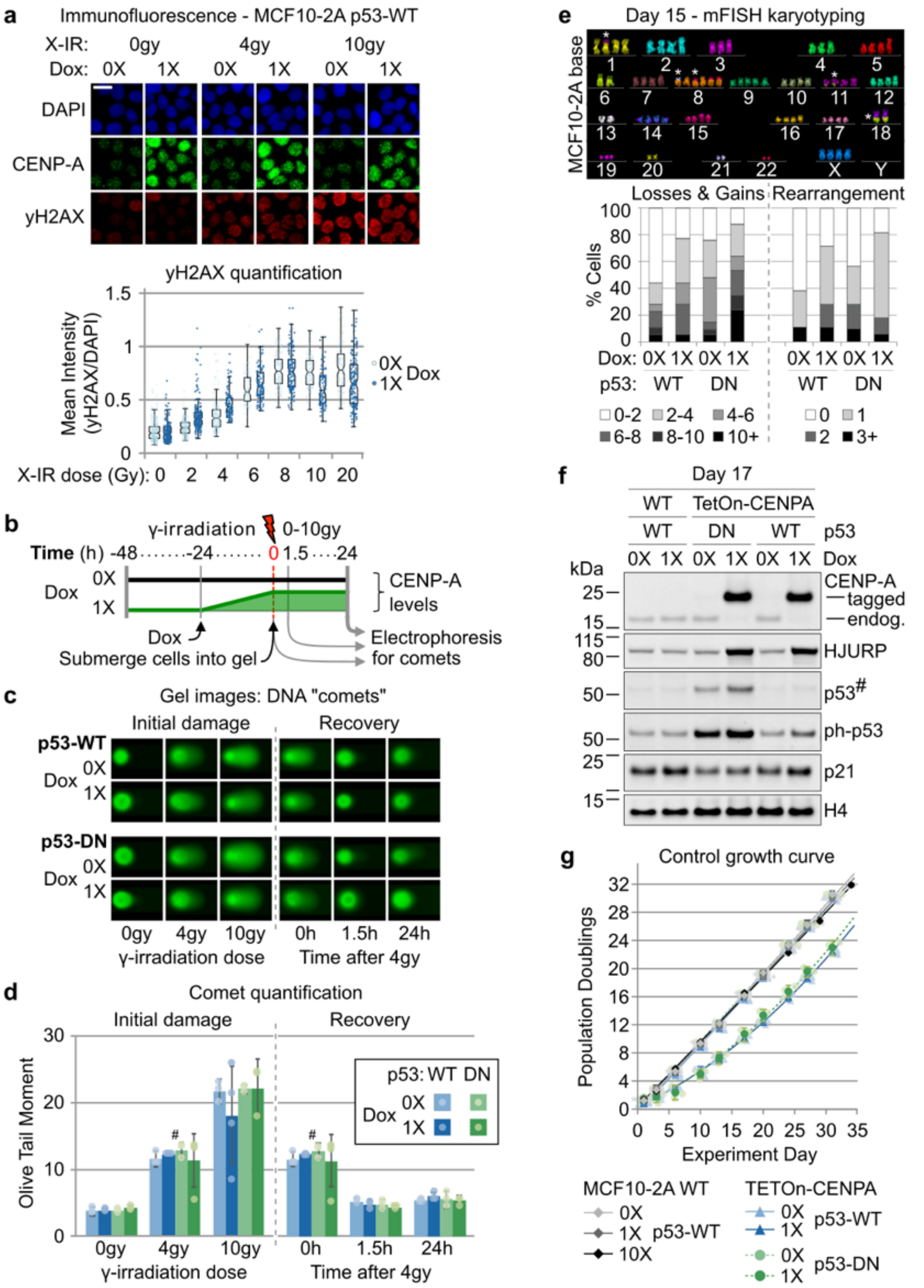

Supplementary Figure 3

Supplementary Figure 3 – Related to Figure 4 and Supplementary Figure 1. Impact of CENP-A overexpression of cell proliferation, DNA damage and CIN

A) γH2AX response of p53-WT cells after X-irradiation with or without CENP-A overexpression. MCF10-2A *TetOn-CENPA-FLAG-HA* cells with empty vector (p53-WT) were plated in culture dishes containing collagen/fibronectin-coated glass cover slips and then treated with 10 ng/ml Dox or no Dox control for 24h. Cells were then irradiated by X-ray generator with 0-20 Gy and immediately fixed for immunofluorescence with γH2AX and CENP-A antibodies. Top: representative max intensity projection images from Z-stack for cells with (+) or without (-) 10 ng/ml Dox after 0, 4 or 10 Gy X-irradiation. Scale bar = 20 μm. Bottom: quantification of mean γH2AX/DAPI intensity per nucleus for each condition (ImageJ). Center lines show the medians; box limits indicate the 25th and 75th percentiles as determined by R software; whiskers extend 1.5 times the interquartile range from the 25th and 75th percentiles. Light blue = No Dox. Dark blue = 1X (10 ng/ml) Dox. N = >90 for each condition

B) Scheme of comet assays with relative CENP-A protein levels over time for MCF10-2A *TetOn-CENPA-FLAG-HA* cell lines. To test the impact of p53 status and CENP-A overexpression on DNA damage directly, we performed alkaline comet assays after γ-irradiation on cells stably expressing empty vector (p53-WT) or p53-DN, with (+) or without (-) 24h 10 ng/ml Dox.

C) Representative images of comets pertaining to scheme in B.

D) Quantification of DNA damage measured as the product of the comet tail length and fraction of DNA in tail (olive tail moment) for each condition pertaining to scheme in B and representative images in C. Plots show mean with 95% confidence interval for the mean olive tail moment from three independent experiments (circles). [#]Same data displayed for 4 Gy 0h treatment shown in both plots for comparison.

E) Quantification of chromosomal abnormalities from mFISH karyotypes in MCF10-2A *TetOn-CENPA-FLAG-HA* cells with either empty vector (p53-WT) or dominant-negative p53 (p53-DN) grown continuously for 15 days without Dox (0X) or with Dox (1X, 10 ng/ml). Top: Representative Metafer automated image of chromosome spread from MCF10-2A *TetOn-CENPA-FLAG-HA* cells, empty vector (p53-WT) without Dox. Asterisks denote baseline chromosome rearrangements that were present in most p53-WT no Dox spreads. Bottom: Stacked bar plots showing percentage of binned counts from at least 17 metaphase spreads per condition pertaining to legends below. Left: Losses and gains of chromosomes per cell determined as the sum of the absolute difference from the mode for each chromosome of the p53-WT no Dox control. Right: New chromosome rearrangements per cell were determined as the total number of structural chromosomal anomalies observed per spread, excluding the ones that were common amongst the p53-WT no Dox spreads.

F) Western blot of TCEs corresponding to Day 17 of growth curve in G for 0X and 1X Dox conditions. Load corresponds to ~50000 cells each. Primary antibodies are indicated on the right. [#] = high sensitivity ECL exposure. H4 used as loading control.

G) Growth curve, as in Figure 4G, but including the non-inducible parental (MCF10-2A WT) cells. Growth curves of MCF10-2A *TetOn-CENPA-FLAG-HA* cells from 0X and 1X Dox (Figure 4G) shown for comparison. Legend: Non-inducible, grey diamonds; p53-WT, blue triangles; p53-DN, green circles. Large dots show mean and 95% confidence interval while smaller dots show individual replicates. Increasing darkness of fill corresponds to increasing Dox 0X, 1X (10 ng/ml) or 10X (100 ng/ml).

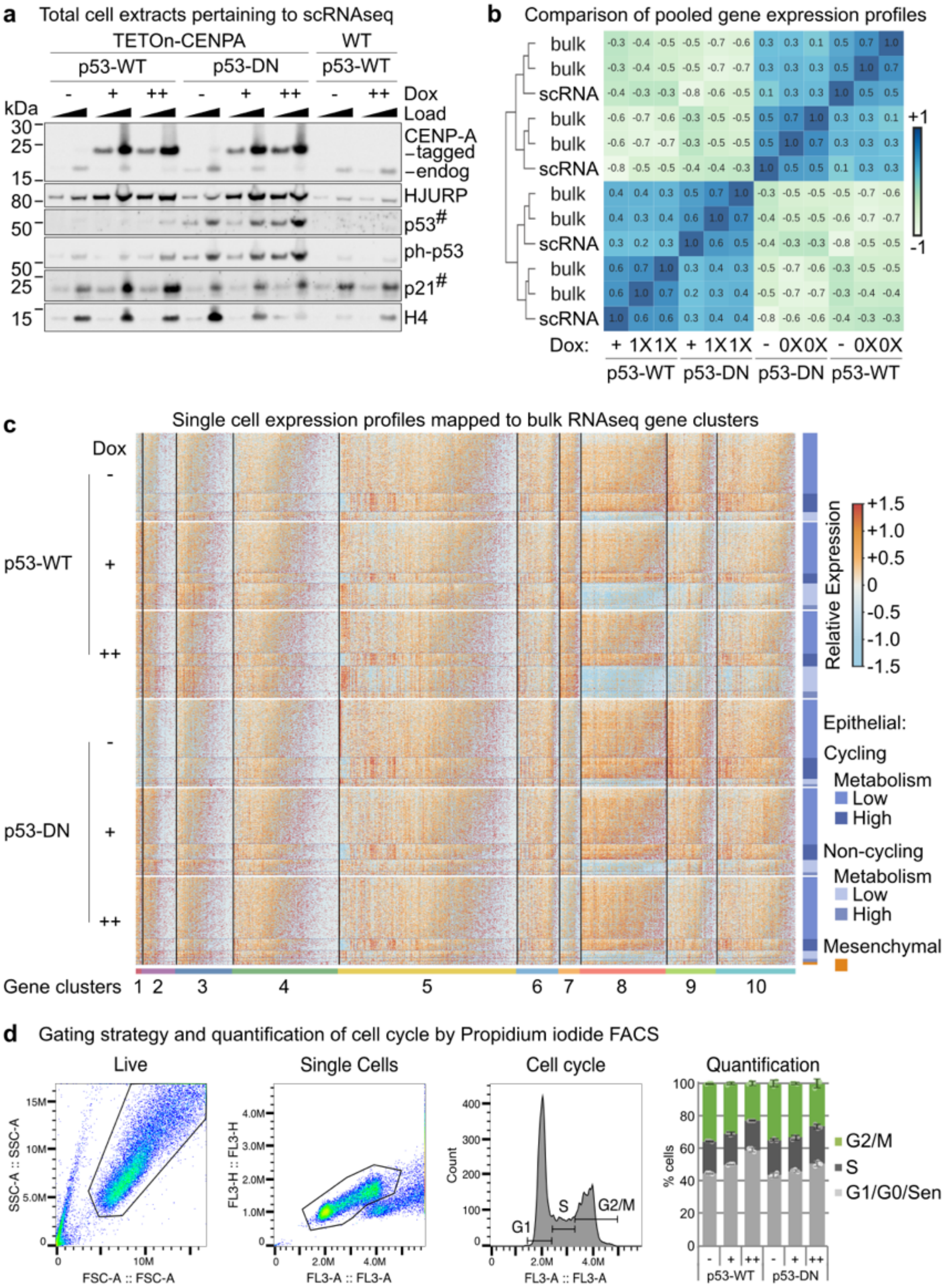

Supplementary Figure 4

Supplementary Figure 4 – Related to Figures 4, 5, 6 and Supplementary Figure 5. Single-cell RNA-seq results compared to Western, bulk RNA-seq, and FACS

A) Western blot using total cell extracts of aliquots from the same samples used for scRNA-seq (see Figures 5 and 6), plus an MCF10-2A non-inducible (WT) parental control cells after long-term exposure to Dox. All cells passaged in parallel for 69 days, or 55 days in the case of the parental line, with either no Dox (-), no Dox followed by 24h Dox (+), or continuous Dox exposure (++) at a concentration of 10 ng/ml Dox. Primary antibodies are indicated on the right. Load: 1x, 3x; where 1x = ~16700 cells. # = high sensitivity ECL exposure. H4 used as loading control.

B) Heat map: clustering of pooled scRNA-seq and bulk RNA-seq samples from comparable experimental conditions (no Dox; 24h 10 ng/ml Dox) based on Pearson correlation among gene expression profiles. Left: row dendrogram showing the clustering of the samples based on average linkage. Corresponding p53 status and Dox treatment indicated on the X-axis (+ or 1X = 10 ng/ml; - or 0X = no Dox; for scRNA-seq and bulk RNA-seq respectively). Positive correlations (blue) and negative correlations (white) are indicated by the colour gradient.

C) Heat map of single-cell expression profiles according to bulk RNA-seq DEG clusters (1-10, labeled at the bottom). 300 cells were randomly sampled per experimental condition. Each row represents a single cell, ordered by experimental condition (indicated on the left) and scRNA-seq cell cluster (indicated on the right, see Supplementary Figure 5A and Table S2 for cell cluster identification). Within each cluster, cells are sorted based on total counts. Each column represents a DEG identified in the bulk RNA-Seq results, ordered by gene cluster and sorted by average expression across all cells. The colour gradient is proportional to the relative expression level across individual cells, from low (blue) to high (orange).

D) Cell cycle quantification by Fluorescence-Activated Cell Sorting (FACS) using propidium iodide-stained MCF102-A Tet-On CENPA-FLAG-HA p53-WT and p53-DN cells. All cells passaged in parallel for 105 days with either no Dox (-), no Dox followed by 24h Dox (+), or continuous Dox exposure (++) at a concentration of 10 ng/ml Dox, then fixed with ethanol. Left three plots show gating strategy. Right: Stacked bar plot shows mean and 95% confidence interval for one experiment performed in triplicate (circles). Green = 2N population (G2/M), Dark grey = S phase, Light grey = 1N population (G1/G0/Senescent), according to the Dean-Jett-Fox cell cycle algorithm with FlowJo software.

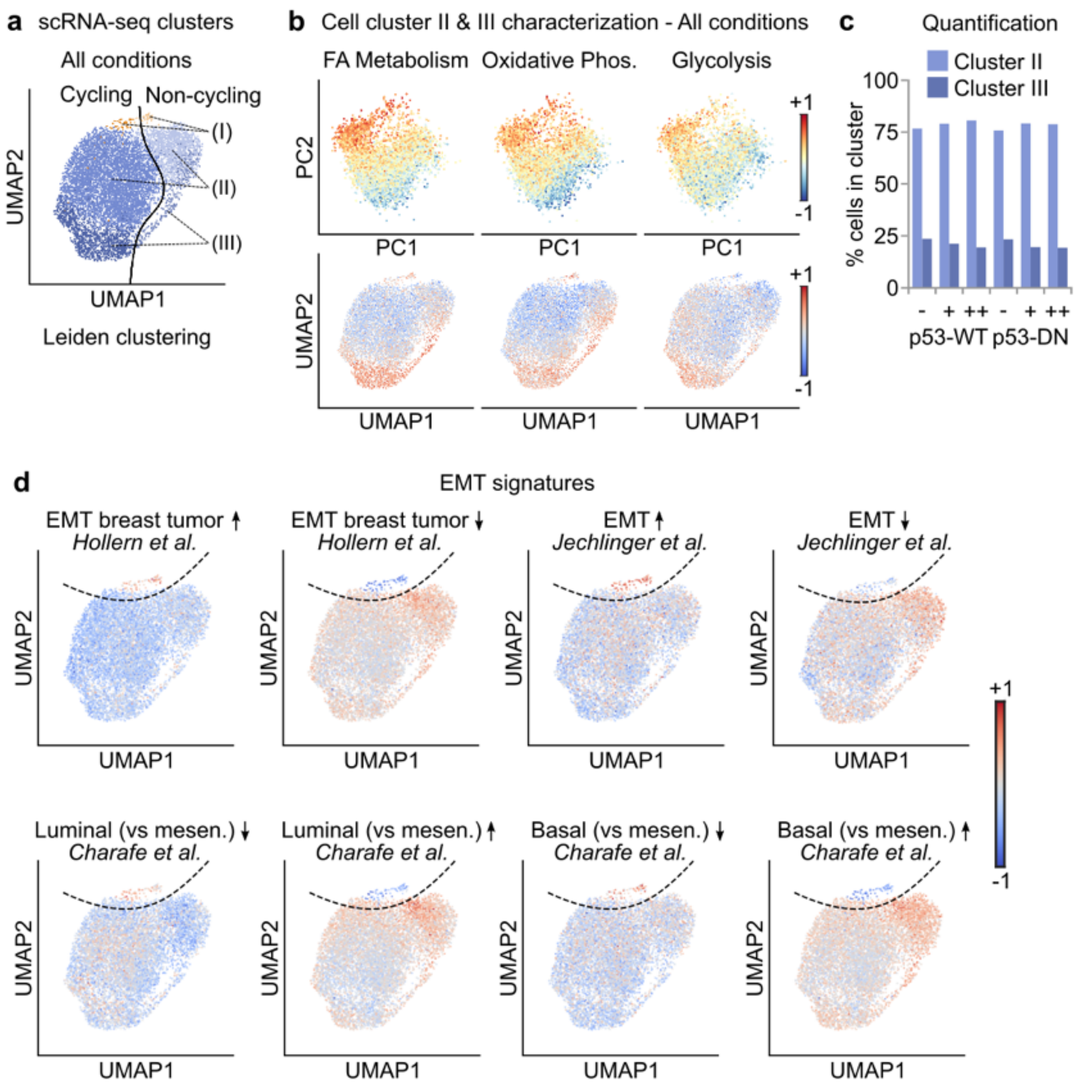

Supplementary Figure 5 – Related to Figures 5 and 6, Supplementary Figure 4, and Supplementary Table 2. Single-cell RNA-seq epithelial cell clusters and EMT signatures

A) UMAP plot of scRNA-seq data pertaining to scheme in Figure 5A, showing three main clusters of cells based on Leiden clustering after cell cycle correction. Cluster I (orange), Cluster II (light blue), and Cluster III (dark blue), are sub-divided as either Cycling (dark) or Non-cycling (pale), according to the previous analysis in Figure 5. See Table S2 for gene markers associated with each cluster, ranked by one-vs-all logistic regression. Cluster I is negative for epithelial markers (e.g. *CDH1*, *EPCAM* and *KRT19*) and shows high expression of mesenchymal genes (e.g. *VIM*, *FN1* and *CDH2*). Clusters II and III are positive for epithelial markers but show differential expression of genes involved in cell metabolism (e.g. *CTSV, AFMID, GLA, ACAT2, CBR4*).

B) Top: Cell-to-cell variability along the second principal component of the scRNA-seq data can be explained by differences in expression of cell metabolism genes. Principal component analysis (PCA) of scRNA-seq experiment. All conditions merged. Each dot represents a single cell on the first (PC1) and second (PC2) principal component, coloured by expression of genes involved in Fatty Acid (FA) Metabolism, Oxidative Phosphorylation (Phos.), and Glycolysis (Hallmark gene sets, MSigDB v6.2). Bottom: As

above, except data plotted in UMAP space. Epithelial clusters II and III show low and high expression of genes involved in cell metabolism processes, respectively.

C) Bar plot showing percentage of scRNA-seq Clusters II (cell metabolism low) and III (cell metabolism high) in A by experimental condition. Proportions are similar for untreated p53-WT and p53-DN cells. CENP-A overexpression (acute or chronic) reduces the proportion of cell metabolism high cells in both cases and the opposite for cell metabolism low cells. We speculate that this represents natural cell-to-cell variation in cell metabolism associated with nutrient availability in the cells, which may be hindered by increased production of CENP-A.

D) UMAP of scRNA-seq samples across all conditions, coloured by the score for selected expression signatures including genes that are up- or down-regulated (arrows) after EMT or in luminal- and basal-like versus mesenchymal breast cancer cell lines (curated gene sets from MSigDB v6.2, see Table S2).

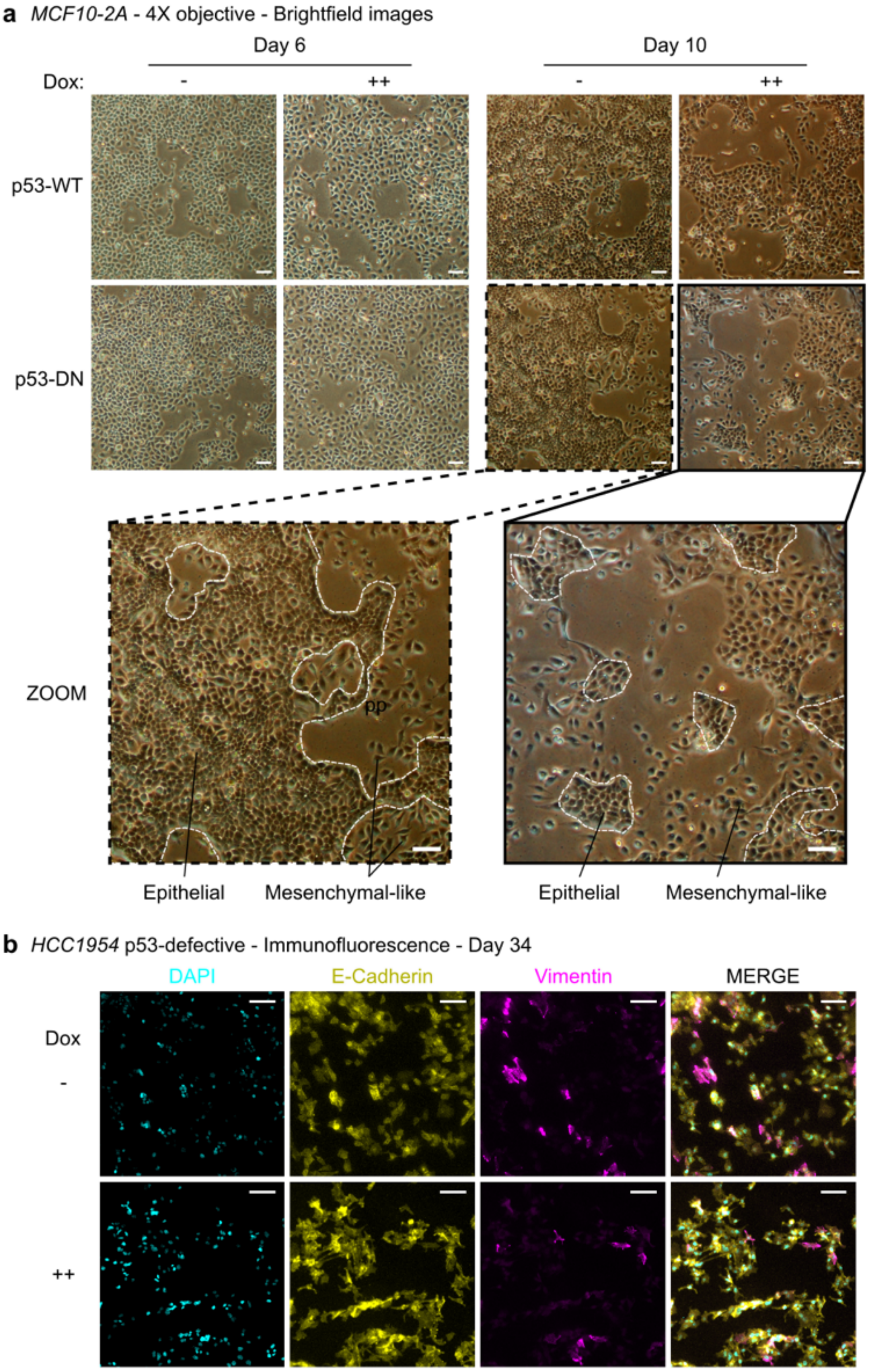

Supplementary Figure 6

Supplementary Figure 6 – Related to Figure 6. Early detection of EMT in p53-DN MCF10-2A cells by brightfield microscopy and lack of EMT in HCC1954.

A) Brightfield images of cells after 6 and 10 days passed in parallel with either no Dox (-) or continuous CENP-A overexpression (++, 10 ng/ml Dox). White dashed lines in zoomed images outline edges of epithelial cell groups. Epithelial cells are characterized by strong cell-cell contacts, compared to cells with mesenchymal-like characteristics (isolated/showing reduced cell-cell contacts and elongated/distorted shape). Images taken of live cells by brightfield microscopy with a 4X objective. Scale bars = 40 µm

B) Assessment of EMT by immunofluorescence of HCC1954 TetOn-CENPA-FLAG-HA cells after 34 days with (++) or without (-) continuous CENP-A overexpression (10 ng/ml Dox). Representative max intensity projections: DAPI (cyan), E-cadherin (yellow, epithelial marker), and Vimentin (magenta, mesenchymal marker). Scale bars = 40 µm. No EMT was detectable in any of the images taken.

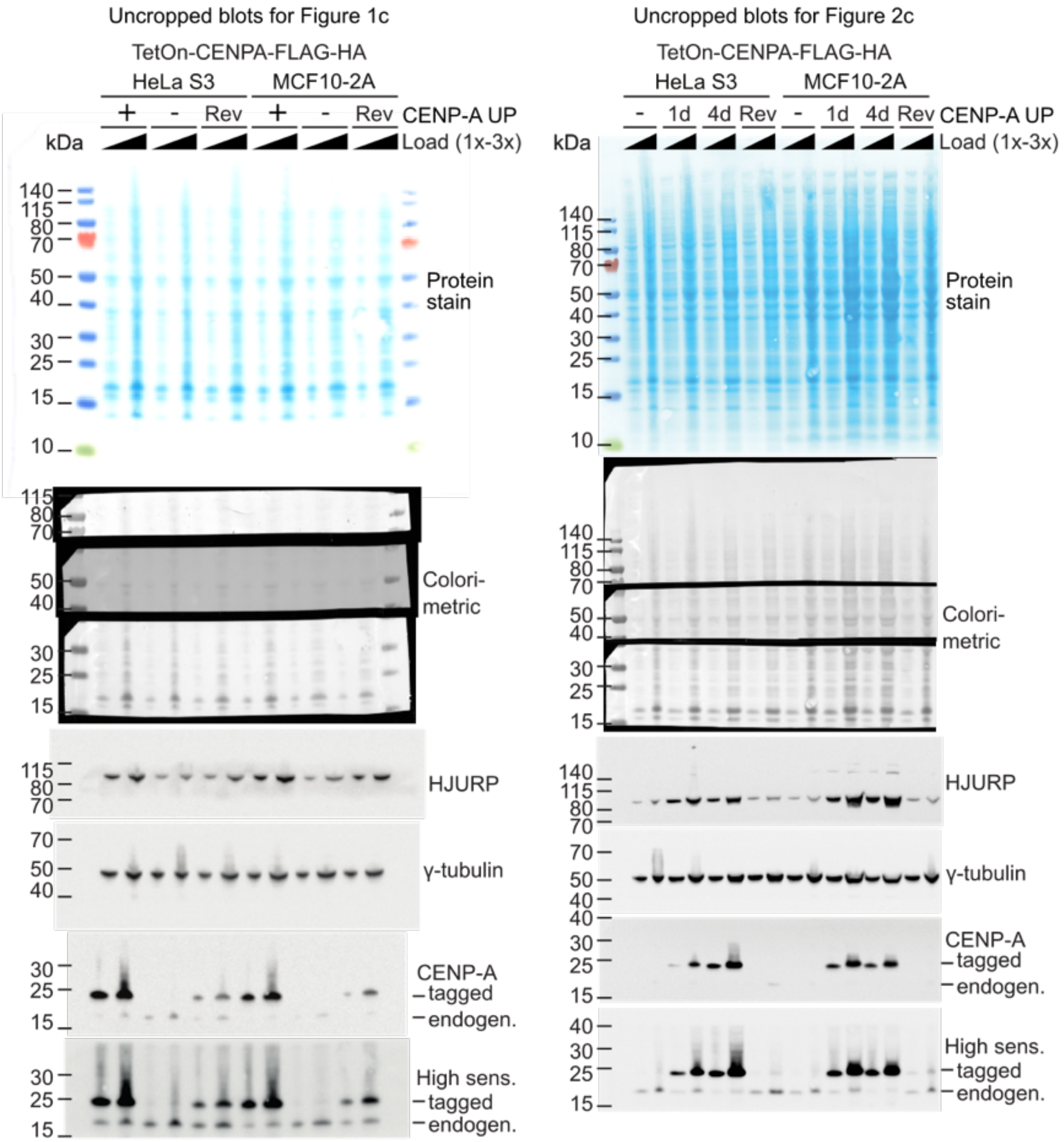

Supplementary Figure 7 – Uncropped Western blots from Figures 1 and 2
Pierce Universal Protein stain, colorimetric images and primary antibodies indicated on the right. High sens. = high sensitivity ECL. Endogen. = endogenous CENP-A. CENP-A UP = growth with (+, 1d, 4d) or without (-) 10ng/ml Dox. Rev = Reversal (growth with 10ng/ml Dox, washout, then growth without Dox).

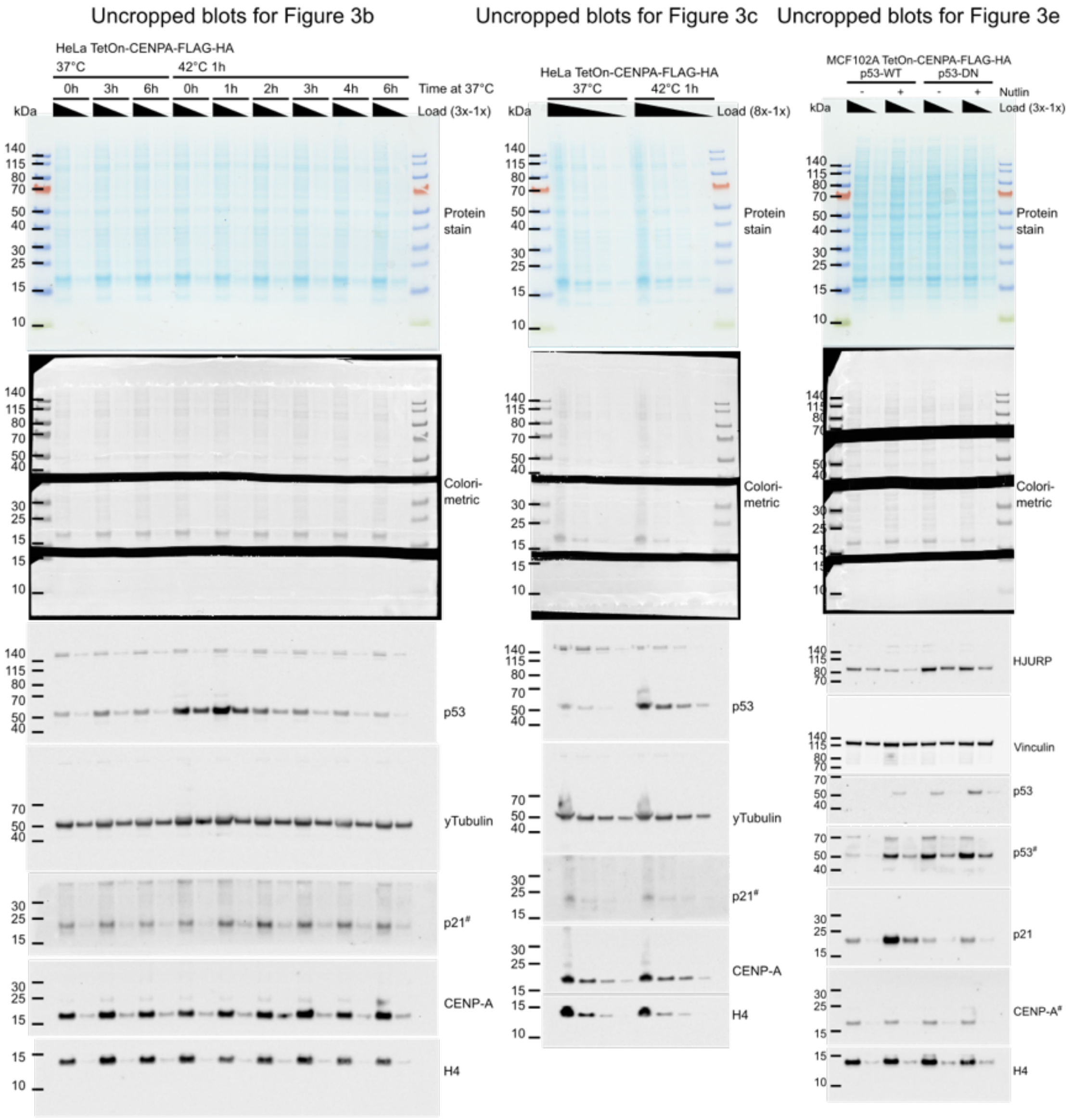

Supplementary Figure 8 – Uncropped Western blots from Figure 3
Pierce Universal Protein stain, colorimetric images and primary antibodies indicated on the right. Antibodies used on the same membrane stained in succession in the order shown. # = high sensitivity ECL.

Uncropped blots for Supplementary Figure 1b

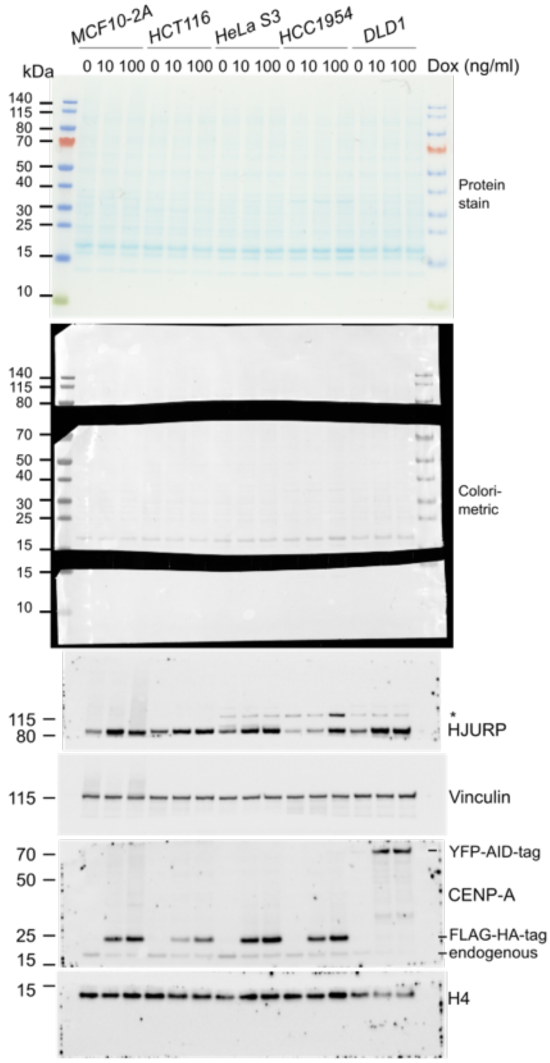

Uncropped blots for Supplementary Figure 1f

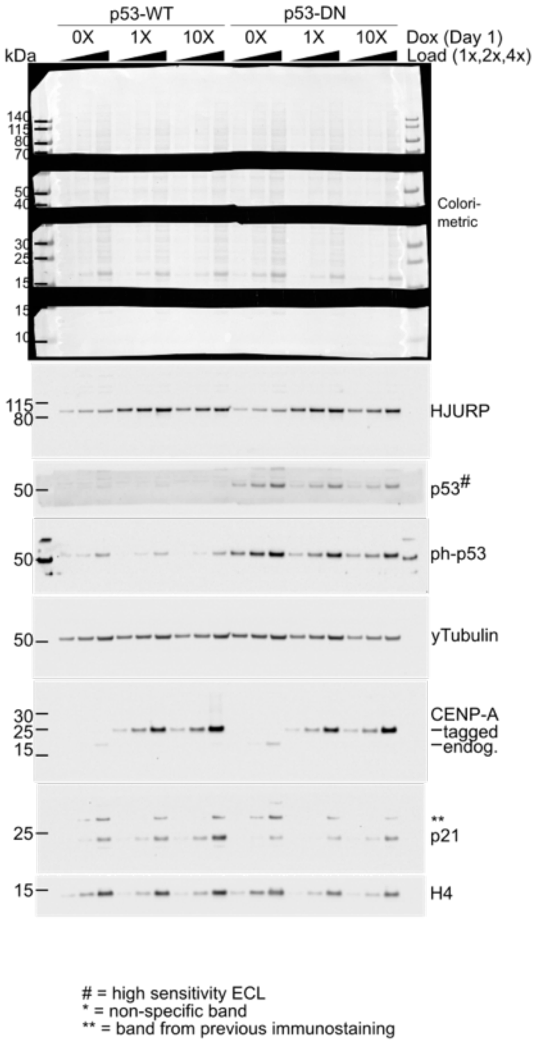

Supplementary Figure 9 – Uncropped Western blots from Supplementary Figure 1
Pierce Universal Protein stain, colorimetric images and primary antibodies indicated on the right. Antibodies used on the same membrane stained in succession in the order shown. # = high sensitivity ECL. * = non-specific band. ** = band from previous immunostaining. Dox used at 10ng/ml (1X) or 100ng/ml (10X).

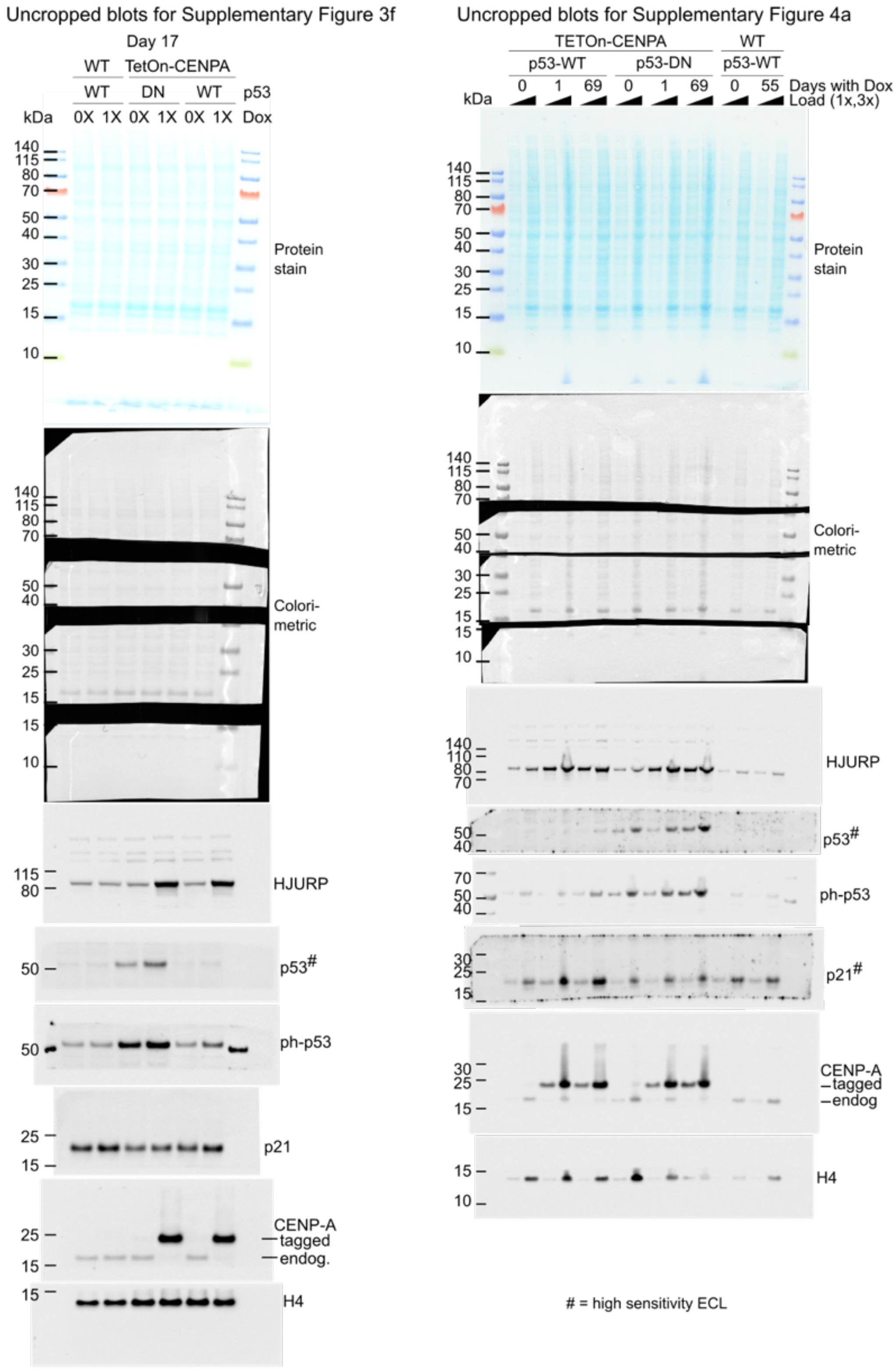

Supplementary Figure 10 – Uncropped Western blots from Supplementary Figures 3 and 4

Pierce Universal Protein stain, colorimetric images and primary antibodies indicated on the right. Antibodies used on the same membrane stained in succession in the order shown. # = high sensitivity ECL. Dox used at 10ng/ml (1X).
