# Supplementary material for: CENP-A overexpression promotes distinct fates in human cells, depending on p53 status
Source: Commun Biol. 2021 Mar 26;4:417. doi: 10.1038/s42003-021-01941-5 (PMC7997993; doi:10.1038/s42003-021-01941-5)
Supplement: Supplementary file 2 — Description of Additional Supplementary Files [file 42003_2021_1941_MOESM2_ESM.pdf]

Description of Additional Supplementary Files

File name: Supplementary Data 1

Description: Source data
Sheet 1: Table of Contents Sheet 2: Source data for colony formation assays (Figures 2 and 3, Supplementary Figure 1) Sheet 3: Source data for micronuclei quantification (Figure 4) Sheet 4: Source data for growth curves (Figure 4 and Supplementary Figure 3) Sheet 5: Source data for beta-galactosidase senescence assay quantification (Figure 5) Sheet 6: Source data for EMT quantification by immunofluoresence (Figure 6) Sheet 7: Source data for Western blot quantification (Supplementary Figure 1) Sheet 8: Source data for RT-qPCR quantification (Supplementary Figure 2) Sheet 9: Source data for yH2AX quantification by immunofluorescence (Supplementary Figure 3) Sheet 10: Source data for comet assay quantification (Supplementary Figure 3) Sheet 11: Source data for mFISH quantification (Supplementary Figure 3) Sheet 12: Source data for cell cycle FACS quantification (Supplementary Figure 4)

File name: Supplementary Data 2

Description: - Related to Figures 4 and S2. Bulk RNA-seq data analysis of MCF10- 2A cells after CENP-A overexpression, switch of p53 status, and X-irradiation.
Sheet 1: Contents & design table Sheet 2: Differential expression analysis of CENP-A overexpression (OE via Dox) effect, p53 effect (p53-DN), and/or Xirradiation (rad) Sheet 3: TMM-normalized counts (excluding non-inducible parental control) Sheet 4: TMM-normalized counts (including control) Sheet 5: All genes ranked according to CENP-A overexpression effect (OE via Dox) based on fold change and p-value Sheet 6: Gene Set Enrichment Analysis (GSEA) by overall CENP-A overexpression effect (OE via Dox) - KEGG pathways, WebGestaltR v0.4.2 Sheet 7: Hierarchical clustering of differentially expressed genes via Ward's method Sheet 8: Over-Representation Analysis (ORA) per gene cluster - KEGG pathways, WebGestaltR v0.4.2 Sheet 9: Linear mixed model to test the effect of p53-DN and X-irradiation across all genes in cluster 8 Sheet 10: Linear mixed model to test the effect of CENP-A overexpression at increasing levels of Dox across all genes in cluster 8.

File name: Supplementary Data 3

Description: Related to Figures 6, S4 and S5. Single-cell RNA-seq cell cluster gene markers.
Sheet 1: Contents Sheet 2: Gene expression signatures references Sheet 3: Mesenchymal/EMT high (all) vs rest Sheet 4: Epithelial Cell Metabolism Low (all) vs rest4 Sheet 5: Epithelial Cell Metabolism High (all) vs rest Sheet 6: Mesenchymal/EMT high (cycling) vs rest Sheet 7: Mesenchymal/EMT high (noncycling) vs rest Sheet 8: Epithelial Cell Metabolism Low (cycling) vs rest Sheet 9: Epithelial Cell Metabolism Low (non-cycling) vs rest Sheet 10: Epithelial Cell Metabolism High (cycling) vs rest Sheet 11: Epithelial Cell Metabolism High (non-cycling) vs rest.
