# Supplementary material for: CENP-A overexpression promotes distinct fates in human cells, depending on p53 status
Source: Commun Biol. 2021 Mar 26;4:417. doi: 10.1038/s42003-021-01941-5 (PMC7997993; doi:10.1038/s42003-021-01941-5)
Supplement: Supplementary file 6 — Reporting Summary [file 42003_2021_1941_MOESM6_ESM.pdf]

nature research

Corresponding author(s): Geneviève Almouzni

Last updated by author(s): February 19, 2021

Reporting Summary

Nature Research wishes to improve the reproducibility of the work that we publish. This form provides structure for consistency and transparency in reporting. For further information on Nature Research policies, see our Editorial Policies and the Editorial Policy Checklist.

Statistics

For all statistical analyses, confirm that the following items are present in the figure legend, table legend, main text, or Methods section.

n/a	Confirmed	
☐	☒	The exact sample size (*n*) for each experimental group/condition, given as a discrete number and unit of measurement
☐	☒	A statement on whether measurements were taken from distinct samples or whether the same sample was measured repeatedly
☐	☒	The statistical test(s) used AND whether they are one- or two-sided<br>*Only common tests should be described solely by name; describe more complex techniques in the Methods section.*
☒	☐	A description of all covariates tested
☐	☒	A description of any assumptions or corrections, such as tests of normality and adjustment for multiple comparisons
☐	☒	A full description of the statistical parameters including central tendency (e.g. means) or other basic estimates (e.g. regression coefficient) AND variation (e.g. standard deviation) or associated estimates of uncertainty (e.g. confidence intervals)
☐	☒	For null hypothesis testing, the test statistic (e.g. *F*, *t*, *r*) with confidence intervals, effect sizes, degrees of freedom and *P* value noted<br>*Give P values as exact values whenever suitable.*
☒	☐	For Bayesian analysis, information on the choice of priors and Markov chain Monte Carlo settings
☐	☒	For hierarchical and complex designs, identification of the appropriate level for tests and full reporting of outcomes
☒	☐	Estimates of effect sizes (e.g. Cohen's *d*, Pearson's *r*), indicating how they were calculated

Our web collection on statistics for biologists contains articles on many of the points above.

Software and code

Policy information about availability of computer code

Data collection	Sequencing performed with a NovaSeq 6000 sequencer. Microscope images obtained by MetaSystems (mFISH, comet assays), MetaMorph (Zeiss microscope) or Softworx (Deltavision microscope) software. Western blot images obtained with ImageLab software. FACS data obtained with an Accuri 6 FACS machine
Data analysis	Bulk RNA-seq analyses were carried out with custom Python scripts. pandas (version 0.24.2), NumPy (version 1.16.2), SciPy (version 1.3.1), and scikit-learn (version 0.21.3) libraries were used for data manipulation, statistical analysis and unsupervised learning. matplotlib (version 2.2.4), matplotlib-venn (version 0.11.5) and seaborn (version 0.9.0) were used for plotting and statistical data visualization. R packages were imported into Python using rpy2 (version 2.8.4).<br>Singe-cell RNA-seq analyses were carried out with custom Python scripts using SCANPY (version 1.4.6). BBKNN (version 1.3.9), UMI-tools (version 1.0.0), pandas (version 0.25.0), NumPy (version 1.17.0), scikit-learn (version 0.21.3), matplotlib (version 3.3.0), and seaborn (version 0.9.0).<br>Image analysis performed with ImageJ (immunofluorescence), Isis (mFISH) or MetaSystems (comet assays) software. FACS analyzed with FlowJo v10.

For manuscripts utilizing custom algorithms or software that are central to the research but not yet described in published literature, software must be made available to editors and reviewers. We strongly encourage code deposition in a community repository (e.g. GitHub). See the Nature Research guidelines for submitting code & software for further information.

Data

Policy information about availability of data

All manuscripts must include a data availability statement. This statement should provide the following information, where applicable:

- Accession codes, unique identifiers, or web links for publicly available datasets
- A list of figures that have associated raw data
- A description of any restrictions on data availability

Bulk- and single-cell RNA-seq data have been deposited in ArrayExpress under accession numbers E-MTAB-9867 and E-MTAB-9861, respectively.

Field-specific reporting

Please select the one below that is the best fit for your research. If you are not sure, read the appropriate sections before making your selection.

- [x] Life sciences
- [ ] Behavioural & social sciences
- [ ] Ecological, evolutionary & environmental sciences

For a reference copy of the document with all sections, see nature.com/documents/nr-reporting-summary-flat.pdf

Life sciences study design

All studies must disclose on these points even when the disclosure is negative.

Sample size	Sample sizes for microscopy quantifications were determined according to the number of countable cells within at least three different fields per replicate. More fields were imaged if fewer cells were present than average. For mFISH, sample size was limited by the number of available spreads in some conditions.
Data exclusions	No data was excluded from the analyses. For quality control of scRNA-seq data, cells with abnormal levels of mitochondrial RNA, after adjusting for depth and total number of detected genes, were filtered out via outlier detection. Outliers were detected by covariance estimation (elliptic envelope with 5% contamination) using the number of genes, total counts (log-transformed) and mitochondrial counts (log-transformed) as features for outlier detection. Genes detected in less than 100 cells (after filtering) were also excluded.
Replication	All experiments, with the exception of sequencing and mFISH experiments, were repeated independently at least once, as indicated in the figure legends. All repeats were consistent with the data reported. Number of biological replicates tested are also indicated for each experiment in the figure legends: typically 3 or 6 biological replicates were used for CFA experiments, 2 or 3 for immunofluorescence and senescence quantifications, 1 for Western blots, 3 for proliferation assays, and 2 for bulk RNA-seq. Single-cell RNA-seq was performed in singlet, but were validated by comparison to bulk RNA-seq.
Randomization	For all manual quantifications (CFAs, immunofluorescence, senescence, micronuclei, mFISH), associated images were randomized and de-labeled prior to analysis to aid in blinding the researchers to the experimental conditions.
Blinding	Researchers were blinded to the experimental conditions for all analyses that involved manual quantifications (CFAs, immunofluorescence, senescence, micronuclei, mFISH).

Reporting for specific materials, systems and methods

We require information from authors about some types of materials, experimental systems and methods used in many studies. Here, indicate whether each material, system or method listed is relevant to your study. If you are not sure if a list item applies to your research, read the appropriate section before selecting a response.

Materials & experimental systems

n/a	Involved in the study	
[ ]	[x]	Antibodies
[ ]	[x]	Eukaryotic cell lines
[x]	[ ]	Palaeontology and archaeology
[x]	[ ]	Animals and other organisms
[x]	[ ]	Human research participants
[x]	[ ]	Clinical data
[x]	[ ]	Dual use research of concern

Methods

n/a	Involved in the study	
[x]	[ ]	ChIP-seq
[ ]	[x]	Flow cytometry
[x]	[ ]	MRI-based neuroimaging

Antibodies

Antibodies used	For immunofluorescence: Primary antibodies: CENP-A (3-19) 1:300 (ADI-KAM-CC006-E Enzo Life Sciences), E-cadherin (24E10) 1:200 (3195S CST), γH2AX 1:250 (2577 CST), Vimentin (N-term) 1:100 (5741S Progen). Secondary antibodies: 1:1000 Alexa Fluor donkey anti-mouse IgG (H+L) 488, 1:1000 Alexa Fluor goat anti-rabbit IgG (H+L) 488 or 594, 1:1000 Alexa Fluor goat anti-Guinea Pig IgG (H+L)

594.
For Western blotting: Primary antibodies: CENP-A 1:500 (2186 Cell Signaling Technology (CST)), H4-pan 1:2500 (05-858, Sigma-Aldrich), HJURP 1:300 (HPA008436 Sigma-Aldrich), γTubulin 1:10000 (T5326 Sigma-Aldrich), p53 (1C12) 1:1000 (2524 CST), p21 1:500 (556431 BD Pharmingen), phospho-p53 (Ser15) 1:500 (9284 CST). Secondary antibodies: Jackson ImmunoResearch, 1:10000, donkey anti-mouse or donkey anti-rabbit.

Validation

All primary antibodies were approved for the given application by the manufacturer. Most of our antibodies were characterized in previous publications from our group or others. Additional validations for each antibody were also performed through internal controls shown in the manuscript and/or showed expected molecular weight for Western blotting or characteristic localization for immunofluorescence.

Eukaryotic cell lines

Policy information about cell lines

Cell line source(s)	Lenti-X 293T cells (632180) were purchased from Clontech. All other parental human cell lines originally from ATCC: HeLa S3 (CCL-2.2), MCF10-2A (CRL-10781), T47D (HTB-133), HCC1954 (CRL-2338). Inducible CENP-A overexpression DLD1 cells obtained from Daniele Fachinetti (Institut Curie).
Authentication	HeLa S3 and MCF10-2A cell lines were authenticated by STR profiling (Powerplex 16 HS). The other cell lines were not authenticated.
Mycoplasma contamination	All cell lines tested negative for mycoplasma contamination.
Commonly misidentified lines (See ICLAC register)	No commonly misidentified cell lines were used in this study.

Flow Cytometry

Plots

Confirm that:

- [x] The axis labels state the marker and fluorochrome used (e.g. CD4-FITC).
- [x] The axis scales are clearly visible. Include numbers along axes only for bottom left plot of group (a 'group' is an analysis of identical markers).
- [x] All plots are contour plots with outliers or pseudocolor plots.
- [x] A numerical value for number of cells or percentage (with statistics) is provided.

Methodology

Sample preparation	We stained fixed cells with propidium iodide using FxCycle PI/RNase solution (ThermoFisher Scientific)
Instrument	FACS Accuri 6 machine
Software	FlowJo software (V10.1r5)
Cell population abundance	Minimum 15000 gated cells, all counts provided in Supplementary Data 1
Gating strategy	Excluding cell debris (SSC-A vs FSC-A) and doublets (FL3-A x FL3-H), see Supplementary Figure 4d

- [x] Tick this box to confirm that a figure exemplifying the gating strategy is provided in the Supplementary Information.
